# A continuous sirtuin activity assay without any coupling to enzymatic or chemical reactions

**DOI:** 10.1038/srep22643

**Published:** 2016-03-04

**Authors:** Sabine Schuster, Claudia Roessler, Marat Meleshin, Philipp Zimmermann, Zeljko Simic, Christian Kambach, Cordelia Schiene-Fischer, Clemens Steegborn, Michael O. Hottiger, Mike Schutkowski

**Affiliations:** 1 Department of Enzymology, Institute of Biochemistry and Biotechnology, Martin-Luther-University Halle-Wittenberg, Kurt-Mothes-Strasse 3, 0610 Halle (Saale), Germany; 2 Department of Enzymology, joint research project gFP5, Institute of Biochemistry and Biotechnology, Martin-Luther-University Halle-Wittenberg, Kurt-Mothes-Strasse 3, 0610 Halle (Saale), Germany; 3 Department of Biochemistry, University of Bayreuth, Universitaetsstrasse 30, 95447 Bayreuth, Germany; 4 IVBMB, University of Zurich-Irchel, Winterthurerstrasse 190, 8057 Zurich, Switzerland

## Abstract

Sirtuins are NAD^+^ dependent lysine deacylases involved in many regulatory processes such as control of metabolic pathways, DNA repair and stress response. Modulators of sirtuin activity are required as tools for uncovering the biological function of these enzymes and as potential therapeutic agents. Systematic discovery of such modulators is hampered by the lack of direct and continuous activity assays. The present study describes a novel continuous assay based on the increase of a fluorescence signal subsequent to sirtuin mediated removal of a fluorescent acyl chain from a modified TNFα-derived peptide. This substrate is well recognized by human sirtuins 1–6 and represents the best sirtuin 2 substrate described so far with a k_cat_/K_M_-value of 176 000 M^−1^s^−1^. These extraordinary substrate properties allow the first determination of K_i_-values for the specific Sirt2 inhibitory peptide S2iL5 (600 nM) and for the quasi-universal sirtuin inhibitor peptide thioxo myristoyl TNFα (80 nM).

Reversible acylation of protein lysine residues is one of the most abundant posttranslational modifications (PTMs) involved in several cellular processes like metabolic regulation, cell cycle control and epigenetics^1^,^2^. Lysine acetylation is determined by the enzymatic activity of lysine acetyltransferases and lysine deacetylases. Recent studies detect alternative acylations as *in vivo* PTMs, including propionylations^3^,^4^, succinylations^5^,^6^, malonylations^6^,^7^, glutarylations^8^, crotonylations^9^, butyrylations^3^, 2-hydroxyisobutyrylations^10^, phosphoglycerations^11^ and myristoylations^12^. The generation of these PTMs is not fully understood, but it is evident that some of these acyl-transfers represent spontaneous reactions with acyl-CoAs or acylphosphates as acyl-donors forming stable amide bonds^13^,^14^,^15^,^16^,^17^. Removal of such acyl moieties from lysine side chains is catalyzed by either zinc ion dependent lysine deacetylases or by a conserved family of NAD^+^ dependent lysine deacylases, known as sirtuins. The mitochondrial sirtuin 5 (Sirt5) has over hundred-fold higher catalytic efficiency for succinylated and glutarylated lysine residues as compared to acetylated lysines^7^,^8^,^18^,^19^, whereas Sirt6 prefers long acyl chains, such as myristoylated lysine side chains^20^,^21^. Recently, it has been demonstrated that Sirt4 is able to remove lipoyl and biotinyl residues from lysine side chains both *in vitro* and *in vivo*^22^ while Sirt3 seems to be an *in vivo* decrotonylase, in addition to its established deacetylase function^23^. Furthermore, it is known that Sirt2 exhibits demyristoylase^24^,^25^ and depalmitoylase activity^26^. Sirtuin mediated deacetylations regulate several metabolic processes, such as fatty acid synthesis, glucose homeostasis and stress response^27^. Moreover, sirtuins are involved in diseases like diabetes, cancer and neurodegeneration^27^, making these enzyme attractive targets for pharmacological modulation. However, few compounds for sirtuin inhibition and activation are available, and the unavailability of sensitive and reliable assays also suitable for high-throughput screens has contributed to this lack of modulators^28^. In fact, assay artifacts contributed to a controversy about the general possibility to activate sirtuins, but more recent work involving robust yet time-demanding low-throughput mass spectrometry-based sirtuin assays confirmed the controversial Sirt1 activation and revealed the possibility to activate Sirt5 and Sirt6^29^,^30^.

For the efficient development of sirtuin effectors, reliable and ideally continuous high-throughput assays are indispensable. Several existing, and in most cases discontinuous activity assays (reviewed in^28^,^31^) are based on the separation of products and substrates by HPLC/CE^32^,^33^,^34^, by mass spectrometry^35^,^36^ or spectrophotometric detection of one reaction component^29^,^37^,^38^,^39^. Nevertheless, continuous activity assays are known which couple the sirtuin reaction to either an additional enzymatic reaction^38^,^40^, a chemical reaction such as intramolecular transesterification^41^, an interaction with DNA^42^,^43^ or to fluorescence enhancement by aggregation-induced emission^44^,^45^.

For microtiter plate (MTP)-based assay formats the sirtuin reaction is currently coupled to enzymatic reactions either sensing the released nicotinamide^38^, the remaining NAD^+ ^46 or the deacylated peptide product^40^. One advantage of monitoring sirtuin-mediated release of nicotinamide is the compatibility with any substrate including proteins and also with any type of lysine acylation. However, the enzymatic cascade needed for signal generation, limits the linear range of the assay and makes it more sensitive to interference in compound tests as observed for GW5074, a Sirt5 inhibitor that also affects GDH activity^47^. Hubbard *et al.* substituted the last enzymatic step by a chemical reaction sensing ammonia allowing more accurate but discontinuous activity determinations^29^,^48^.

Assays sensing the deacylated product of the sirtuin reaction utilize the subsite specificity of proteases like Trypsin, which have a strong preference for positively charged side chains in the P_1_-position and thus do not cleave the acylated substrates of the sirtuin-mediated reaction. This principle has been introduced using peptidyl-7-amino-4-methyl-coumarin derivatives^49^.

Subsequent to deacylation of the peptidyl moiety the bond between the C-terminus of the peptidyl moiety and the amino-coumarin derivative is hydrolyzed by the helper protease hereby releasing the highly fluorescent 7-amino-4-methyl-coumarin.

This assay is very sensitive^49^ but makes use of sirtuin substrates with suboptimal K_M_ values and it often has to be performed discontinuously because of the susceptibility of sirtuins to digestion by the helper protease. Appropriate substrates have been synthesized for assaying sirtuin isoforms activity against acetylated^49^, succinylated^50^, glutarylated^8^, adipoylated^8^ or myristoylated lysine residues^51^. The fluorophore replacing the C-terminal peptide part renders these substrates highly artificial and has been reported to cause artifacts in compound tests^28^,^30^,^52^,^53^. Improved substrates for Sirt1 and Sirt2 have been reported using FRET by introducing tetramethylrhodamine as a fluorophor and QSY-7 as a quencher at the N- and C-terminus, respectively, of a p53- derived peptide^37^. For Sirt5 and Sirt6 activity measurements the fluorophore/quencher pair Dabcyl and EDANS was used in a glutamate dehydrogenase derived peptide sequence^54^,^55^. Recently, we were able to show that use of 2-aminobenzoylamides as fluorophores and 3-nitrotyrosines as quenchers in a carbamoyl phosphate synthetase 1 derived peptide derivative allow sensitive detection of Sirt5 activity in a continuous format^56^.

Relatively high amounts (up to 4 μM) of sirtuin have been used in activity assays to correct for suboptimal substrate properties^20^. This limits the applicability of the Michaelis-Menten-equation, which is valid only if enzyme concentration is much lower than substrate concentration. Additionally, due to the high enzyme concentrations, reliable estimation of IC_50_- or K_i_-values is difficult for inhibitors with affinities far below the enzyme concentration.

As previously established, sirtuins 1–6 are able to remove mid-chain acyl residues like octanoyl-, decanoyl- and myristoyl-moieties from lysine side chains in histone H3 derived model peptides^20^,^57^. Recently, using a similar histone H3 peptide substrate, it could be demonstrated that Sirt1-3 are able to remove myristoyl residues from lysine side chains^25^ and Sirt2 represents a very efficient demyristoylase^24^. This fact and inspection of several crystal structures of a myristoylated/thioxo myristoylated peptides in complex with Sirt6/Sirt2 (PDB IDs 3ZG6, 4R8M, 4Y6Q, 4Y6L)^21^,^24^,^57^ prompted us to test if the hydrophobic channel on the surfaces of Sirt6^21^ and Sirt2^24^,^26^,^57^ can accommodate small fluorophores, like 2-aminobenzoylamides, to create a continuous sirtuin activity assay.

If accepted by sirtuins, replacement of one amino acid residue within the TNFα derived substrate by a 3-nitrotyrosine residue as a quencher moiety should yield a peptide derivative increasing its fluorescence subsequent to sirtuin treatment in the presence of NAD^+^(Fig. 1).

## Results

We synthesized peptides derived from the TNFα sequence which are used as model substrates^21^,^24^,^25^ or inhibitors^24^,^25^ for different sirtuin isoforms (Figs 2 and 3). Nosyl-protection at one lysine residue for selective on-resin modification of this side chain and Fmoc-based solid phase peptide chemistry was employed. The peptide **1a** is the best Sirt6 substrate described so far^21^.

**1a** and **2a** were subjected to HPLC-based activity assay to assess their substrate properties and to determine if the quencher moiety is accepted by sirtuin 6. Negative controls without NAD^+^ under identical conditions yielded no conversion of substrates. The kinetic constants uncovered that the replacement of threonine in +1 position of the substrate by the quencher moiety 3-nitrotyrosine did not influence the turnover number and minimally disturbed the apparent affinity to the active site of Sirt6 as reflected by the almost comparable K_M_ values for **1a** (6 μM) and **2a** (17 μM) (Supplementary Fig. S3).

Sirtuins 1–5 were also tested and **1a** was shown to represent a universal sirtuin substrate with k_cat_/K_M_ values in the range of 10 to 50 000 M^−1^s^−1^ (Supplementary Table S2). Therefore, we reasoned that use the fatty acid chain could be utilized as an attachment point for the very small fluorophore 2-amino-benzoylamide. Systematic variation of the distance (number of bonds) between the amide bond on the lysine side chain and the 2-amino-benzoylamide moiety (i.e. **3**, **4**, **4a**) revealed good substrate properties for **3** only (Table 1). Peptide **4** was not a substrate for sirtuins 1 and 3–7 but showed some activity for Sirt2 in an HPLC based end-point activity assay. However, increasing the number of methylene groups to place the fluorophore to a different position yielded improvement in substrate properties for Sirt2 with an optimum for **3**. Further elongation of the spacer resulted in >1000-fold decrease of substrate properties for Sirt2 (Table 1) and complete loss of activity for sirtuin isoforms 1 and 3–6. Substrate **3** represents a quasi-universal sirtuin substrate because it is recognized by isoforms 1–6. The development of **3** resulted in slightly decreased substrate properties for sirtuins 1, 3, 4, 5, and 6 as compared to **1a** but interestingly yielded an improved substrate for Sirt2 with a specificity constant of 176 000 M^−1^s^-1^ representing the best Sirt2 substrate described so far (Table 2). Sirt7 was not able to recognize substrate **3** pointing to structural differences of the hydrophobic channel accommodating the acyl chain.

Sirtuin mediated transformation of **3** into **2b** could be followed directly and continuously (λ_Ex_ = 310 nm, λ_Em = _405 nm) using a fluorescence spectrometer (Supplementary Fig. S5). Without NAD^+^ in the presence of sirtuin enzyme or without sirtuin in the presence of NAD^+^ no significant change in fluorescence signal over time could be observed (Supplementary Fig. S5). This indicated that the observed fluorescence change results directly from sirtuin-mediated deacylation and not from unspecific interactions between NAD^+^ and/or sirtuin and **3**.

The slope of change in fluorescence intensity is linearly dependent on the enzyme concentration (Supplementary Fig. S5). Progress curves at different concentrations were linear below 25% conversion of the substrate. We used a completely converted assay solution (controlled by LC-MS) for the generation of appropriate calibration curves (Supplementary Fig. S9). Additionally, we were able to demonstrate that the activity assay is compatible with 96- and 384-well microtiter plate-based equipment yielding Z´-factors of 0.85 for **3** at 25 μM concentration. Kinetic constants determined with either HPLC based assay or with the assay performed in both MTP fluorescence readers and spectrophotometers yielded comparable results (Supplementary Table S2).

Due to the relatively low k_cat_-values of the known substrates, “classical” sirtuin activity assays are done in timeframes between 30 and 120 min and at enzyme concentrations between 0.5 and 4 μM to generate sufficient signal changes. At these conditions the basic assumption of the Michaelis-Menten-equation 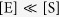 is not valid. Moreover, the high amount of enzyme prevents the correct determination of K_i_-values for sirtuin inhibitors with affinities below half of the enzyme concentrations used. With substrate **3** we were able to follow enzymatic activities down to 10 nM sirtuin concentration (Supplementary Fig. S8). We used a 96-well MTP fluorescence reader for the determination of the K_i_-values for different compounds (Fig. 3) including inhibitors with high affinities to sirtuin isoforms (Table 3).

The first product of the sirtuin reaction, nicotinamide (NAM), is known to be a non-competitive inhibitor with respect to both acylated peptide substrate and NAD^+^ cosubstrate by re-binding to the active site and attacking the sirtuin bound *O*-alkylimidate reforming NAD^+^. For Sirt6 an IC_50_ value of 2.2 mM was reported for NAM indicating that this isoform is not influenced by physiological NAM concentrations^58^

We determined the K_i_-values of NAM for Sirt3 and Sirt6 to be 93 μM and 451 μM, respectively, using NAD^+^ at saturating conditions (Supplementary Fig. S13). Under peptide substrate saturating conditions K_i_-values were found to be 45 μM and 415 μM, respectively (Supplementary Fig. S13). The K_i_-value for NAM was lower than expected for Sirt6, but still higher than for other isoforms. Recently, it was shown that the IC_50_-values for NAM are dependent on the chemical nature of the acyl moiety and that different sirtuin isoforms have different acyl-dependent susceptibilities to NAM inhibition^57^. Our substrate closely resembles the physiological myristoyl substrate hence our value should reflect the sensitivity of this substrate modification. Recently, compounds Quercetin and Ex-527 were reported as Sirt6 inhibitors with inhibition of enzymatic activity of 52% and 56%, respectively, if used at 200 μM concentration^58^. We determined K_i_-values for these two small molecules and found considerable non-competitive inhibition with respect to the peptide substrate (Table 3). The cyclic peptide derivative S2iL5, containing a trifluoroacetylated lysine side chain as a warhead for inhibition of sirtuin catalysis^59^, was claimed to be a Sirt2 specific inhibitor with affinities to the active site in the low nanomolar range as determined by isothermal calorimetric measurements^60^. Using **3** as substrate the determined K_i_-value is 560 nM and the cyclic inhibitor behaved non-competitive for the peptide substrate (Supplementary Fig. S17). Replacement of the amide bond formed by the acyl chain and the ε-amino function of the lysine side chain by a thioxo amide bond transforms substrates into extremely slow substrates/inhibitors by generation of a stalled intermediate resembling sirtuin bi-substrate inhibitors^61^,^62^,^63^. Thioxo myristoylated and shortened derivatives of **1** were shown to be cell permeable inhibitors for Sirt6 with remarkable cross-reactivity to Sirt1-3 and reported IC_50_ values in the single digit micromolar range^25^. Due to the high sirtuin concentration used in this enzymatic assay (i.e. 1 μM Sirt6) we wondered if these values are too high, not properly reflecting the K_i_. We determined the K_i_-values of **8** for sirtuins 2, 3, and 6 using substrate **3** and 96-well-based readout (Table 3) and determined higher affinities to the sirtuins especially for Sirt2 with K_i_-value of 80 nM. We were able to determine these K_i_-values because in our case the enzyme concentration was about 100 times lower as compared to the assay proposed by He *et al.*^25^. Additionally, Sirt3 showed high affinity to **8**. We were not able to determine the K_i_-values if we pre-incubate the enzymes Sirt2 and Sirt3 (10 nM) with different concentrations of **8** in the presence of NAD^+^ for 30 min enabling formation of the “stalled” intermediate without competition with the substrate peptide. Starting the reaction by addition of **3** we observed complete inhibition with low nanomolar concentrations of **8** demonstrating that the pre-formed bi-substrate inhibitor has affinities to Sirt2 and Sirt3 in the very low nano- or picomolar range. The resulting non-competitive inhibition against peptide substrate was in accordance with the suggested model of bi-substrate like inhibitors for thioxo acylated derivatives (Supplementary Fig. S15).

Identification of small molecule modulators of sirtuin activity using **3** could be hampered by absorbance/fluorescence of the effectors in the range between 320 nm and 400 nm. Consequently, the known sirtuin activity modulator resveratrol could not be analyzed because of the high extinction coefficient in that range. Using HPLC-based activity assay we found no significant influence of resveratrol on Sirt1 mediated deacylation of **3** (Supplementary Fig. S4) To be able to analyze small molecules with absorption/fluorescence in the range of 2-aminobenzoylamide fluorescence, we exchanged the 2-aminobenzoylamide fluorophore by (4-*N*,*N*-dimethylamino-1,8-naphthalimido)-acetamide resulting in derivative **6** with fluorescence excitation at 471 nm^64^. Surprisingly, this fluorophore could be used in combination with the 3-nitro-L-tyrosine quencher despite non-optimal overlap of the spectra. Compound **6** was not a substrate for sirtuins 1, 3, 5 and 6 as determined by HPLC-based assays and was a weak substrate for Sirt2 with an about 500-fold lower k_cat_/K_M_-value as compared to **3**. Interestingly, Sirt4 recognized **6** better than **3** resulting in an about 10-fold improved K_M_-value (Supplementary Table S2) indicative of differences in the flexibility of the hydrophobic channel accommodating the acyl chain between Sirt4 and the other sirtuin isoforms. However, these results showed that the development of substrates with different spectral properties is possible enabling the simultaneous detection of enzymatic activity using substrate mixtures. We analyzed kinetics for Sirt2 and Sirt4 using a mixture of substrates **3** and **6** using 290 nm for excitation of both fluorophores and recording fluorescence spectra over time (Supplementary Fig. S10 and S11). Furthermore development of isoform selective substrates should be possible by systematic variation of the size and position of the fluorophore in the acyl side chain.

**Figure 1:**
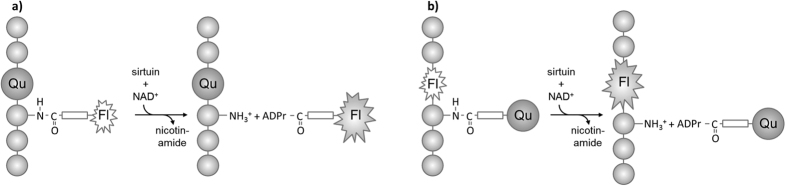
(**a**) Sirtuin-mediated deacylation reaction transfers fluorescently labeled acyl residues from lysine side chain to ADP-ribose. (**b**) Sirtuin-mediated deacylation reaction transfers quencher-containing acyl residue from lysine side chain to ADP-ribose. In both cases sirtuin activity causes an increase in the fluorescence signal. (Fl – fluorophore, Qu – quencher, ADPr – ADP ribose)

**Figure 2:**
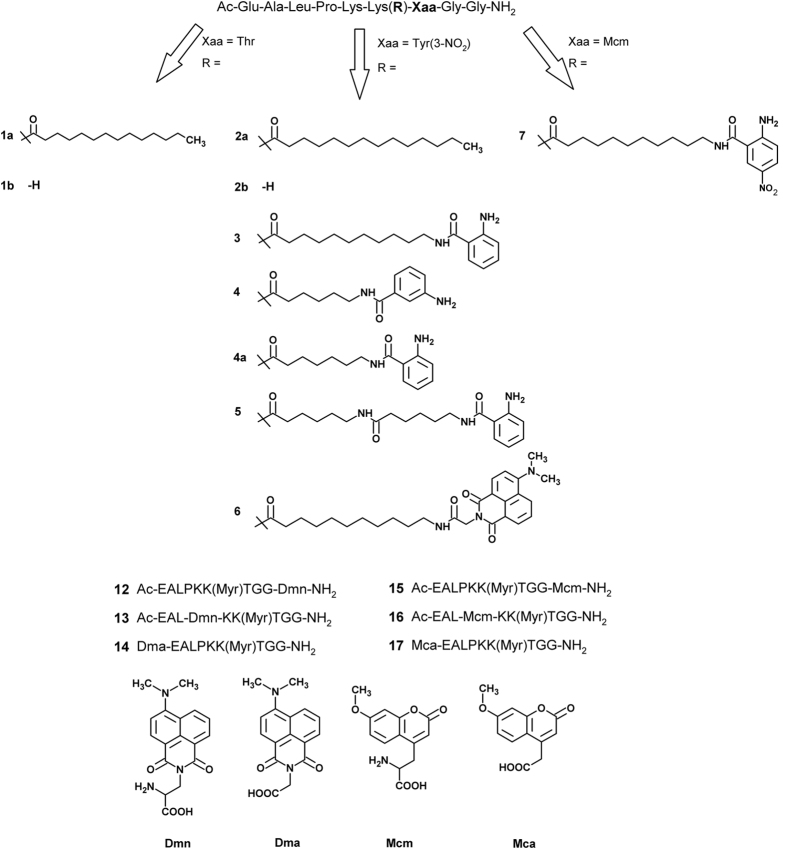
Structures of synthesized substrates.

**Figure 3:**
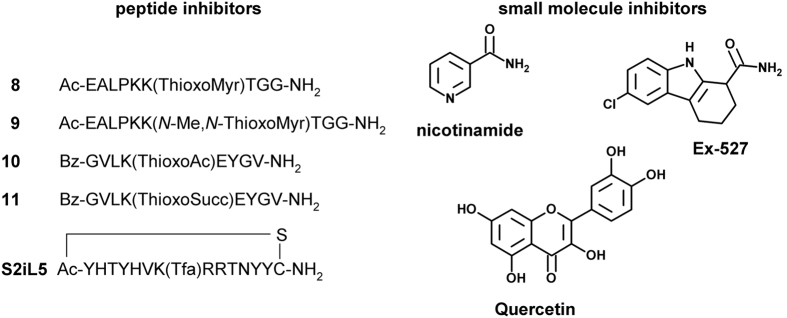
Structures of inhibitors.

Because of the obvious limitations for most of the sirtuins in accommodating bulkier fluorophores, we decided to create a small quencher moiety at this position closely related to the well-recognized 2-amino-benzoylamide residue. Addition of a nitro function in *para*-position to the amino-group of the 2-amino-benzoylamide moiety generated a very efficient quencher for more bulky fluorophores like 7-methoxy-coumaryl-L-alanines (Mca) or even (4-*N*,*N*-dimethylamino-1,8-naphthalimido)-L-alanines (Dma) (data not shown). We speculated that sirtuins are less sensitive for modifications within the peptide sequence and synthesized several derivatives of **1a** characterized by substitutions of residues in different positions relative to the myristoylated lysine by either Dma (**12**, **13**) or Mca (**15**, **16**). Additionally, we attached the fluorophores to the *N*-terminus in form of appropriately substituted acetyl residues (**14**, **17**). Analysis of sirtuin 2, 3, and 6 activity against these substrates using an HPLC-based assay revealed that all peptides are substrates but only **13** and **16** are well recognized (Supplementary Table S1). For solubility reasons (data not shown) we decided to combine the 7-methoxy-coumaryl-L-alanyl-residue with the 5-nitro-2-amino-benzoylamide quencher moiety resulting in **7**. The substrate properties of **7** for Sirt2 and Sirt4 are superior to substrates described in the literature and similar to **3**, demonstrating that fluorophore and quencher positions could be switched without influence on substrate properties (Supplementary Table S2 and Fig. 1).

Inspection of the published crystal structure of Sirt6 in complex with the myristoylated H3K9 substrate (PDB ID 3ZG6) and the respective electron density maps revealed that both conformations of the amide bond between the fatty acid and the lysine side chain amino function could be fitted, but the published coordinates are given in a conformation resembling the *cis* conformation of peptide bonds^21^. In the recently reported structures of Sirt2 complexed with a thioxo myristoylated inhibitor closely related to **8** (PDB ID 4Y6Q) or a thioxo myristoylated peptide derived from Histone H3 (PDB IDs 4Y6L and 4R8M) the conformation of the thioxo amide bond was in *trans* conformation^24^,^57^ which is the preferred conformation of secondary amide/thioxo amide bonds in aqueous solutions. We synthesized **8** in order to analyze if there is any isomer-specificity during binding to the active site of Sirt2 and Sirt6. C*is*/*trans* isomerizations of secondary amide bonds are too fast compared to the time needed for “classical” sirtuin activity assays preventing such analyses. Our assay allowed enzymatic measurements within short time and the isomerization of thioxo amide bonds is slower at lower temperatures^65^. Moreover, the UV-absorption of the π-π* transition for the *cis* conformer of thioxo amide bond is slightly red-shifted enabling determination of *cis*/*trans* isomerization rates using UV-spectroscopy^66^. We determined the isomerization rate for **8**, **10** and **11** at different temperatures subsequent to increasing the *cis* content in the photo-excited state using UV-light (Supplementary Figs S19–33)^66^,^67^. The re-equilibration to the ground state (nearly 100% *trans*-conformation) could be followed using UV-spectroscopy at 260 nm yielding activation parameters (Supplementary Figs S19–33). In order to analyze isomer-specific inhibition of Sirt2 by **8** we optimized the assay conditions to measure the enzymatic activity using **3** without significant *cis*/*trans* isomerization during the assay. The *cis* content of **8** is 2.5% in assay buffer and up to 25% in the photo-excited state as measured by HPLC (Supplementary Fig. S34). We determined the rate of re-equilibration for **8** and calculated the resulting *cis* content subsequent to different times of darkness (Supplementary Table S3). This setup enabled the determination of inhibition of Sirt2 mediated deacylation of **3** at different *cis* contents of **8** ranging from 2.5% to 25%. We found no significant difference in inhibitory effect depending on the *cis* content of **8** indicating an unexpected plasticity of the active site of Sirt2 to accommodate both conformations with similar affinities. This result indicated that the Sirt2/myristoyl-peptide complexes, which were modeled as in *trans* conformation^24^,^57^ would also be compatible with *cis*. IC_50_-values of Sirt6 were determined as 1.06 ± 0.12 μM and 1.87 ± 0.18 μM for 2.5% and 25% *cis* isomer of **8**, respectively, pointing to a small preference for the *cis* conformation of the amide bond (Supplementary Fig. S36). Nevertheless, because of the suboptimal substrate properties of **3** for Sirt6 the assay duration was 30 min which allows significant re-equilibration of the photo-induced change of the *cis*/*trans* equilibrium. Therefore, we introduced an additional methyl group at the lysine nitrogen resulting in a tertiary thioxo amide **9** which is an inhibitor with similar affinities to the active site of Sirt6 as compared to **8** (Supplementary Table S4). The rate constant for the *cis*/*trans* isomerization of the tertiary thioxo amide bond of **9** (5.4 × 10^−4^ s^−1^ at 20 °C) was much slower than that of the secondary thioxo amide bond of **8** (Supplementary Fig. S35). HPLC analyses revealed a *cis* content of about 50% and there was no change detectable subsequent to photo-excitation at the π-π* transition of the tertiary thioxo amide bond. We tested several different organic solvents to change the *cis* content but found no sufficient differences (Supplementary Table S5). Therefore, the two isomers were separated by HPLC at low temperatures (4 °C).

**Figure 4:**
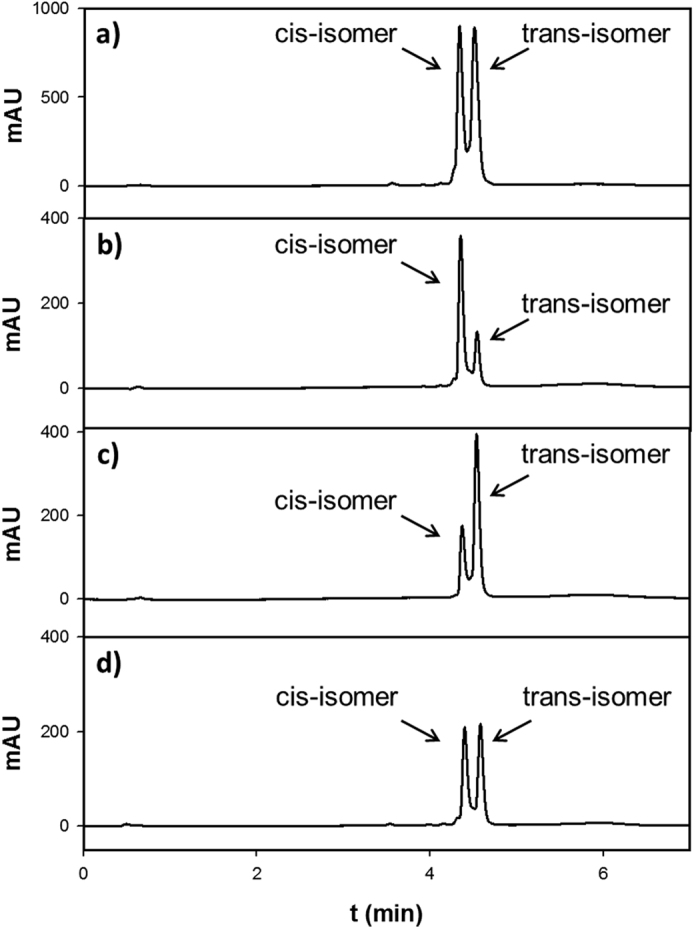
Enrichment of *cis* and *trans* isomers of **9** using HPLC. (**a**) 50 mM solution of **9** equilibrated at RT for 24 h. (**b,c**) Fractions collected by HPLC. (**d**) Aliquot of fraction (**b**) was equilibrated at 20 °C for 24 h.

We were able to enrich the faster migrating *cis* isomer to 72.4% and the *trans* isomer to 70.3% (Fig. 4). The frozen isomers (−70 °C) were stable for several days (Supplementary Fig. S39).

Determination of inhibition of Sirt6 by **9** using samples with different *cis* content showed minimal preference for the *cis* isomer (IC_50_ values of 0.6 μM and 1.7 μM for 72.4% and 29.7% of *cis* of **9**, respectively Supplementary Fig. S38). These results again demonstrated the plasticity within the active site of sirtuins, at least for Sirt6, enabling both isomers to bind with similar affinities. Inspection of the electron density maps of PDB 4R8M and 3ZG6 suggest that there is sufficient space around the lysine side chain amide/thioxo amide bond to fit both isomers. Recently, Sirt6 coordinates of 3ZG6 were re-refined by Denus lab and it was established that the myristoylated peptide should be in a *trans* conformation regarding the amide bond between the lysine side chain and the acyl moiety^57^.

Here we present a continuous sirtuin activity assay allowing convenient measurement of highly accurate data. The sensitivity of the activity assay enables the reliable determination of K_i_-values for inhibitors with affinities below 100 nM. Because of the demonstrated compatibility with 384-well MTP readout we expect that this assay principle will find widespread application in drug discovery projects. Additionally, the superior substrate properties of **3** allow the investigation of isomer specificity in the binding of inhibitors to the active site of sirtuins enlarging the portfolio of tools in sirtuin research.

## Methods

### Chemicals and general methods

All chemicals were purchased from Sigma (Saint Louis, USA) if not denoted otherwise. Rink amide MBHA resin was obtained from Iris Biotech (Marktredwitz, Germany). 9-fluorenylmethoxy-carbonyl- (Fmoc) protected amino acid derivatives and *O*-(Benzotriazol-1-yl)-*N*,*N*,*N*′,*N*′-tetramethyluronium hexafluorophosphate (HBTU) were purchased from Merck (Darmstadt, Germany). Trifluoroacetic acid (TFA) was obtained from Roth (Karlsruhe, Germany).

For HPLC separations solvents consisting of water (solvent A) and ACN (solvent B), both containing 0.1% TFA, were used. Analytical runs were performed on an Agilent 1100 HPLC (Boeblingen, Germany) with a quaternary pump, a well-plate autosampler and a variable wavelength detector. Separations were performed on a 3.0 × 50 mm reversed phase column (Phenomenex Kinetex XB C-18, 2.6 μm) with a flow-rate of 0.6 mL/min. A Merck-Hitachi High Speed LC system (Darmstadt, Germany) with a Merck Hibar Li Chrospher® RP-8 column (250–25 mm, 5 μm) was used for preparative separations (flow-rate: 8 mL/min). Eluted compounds were analyzed by MALDI mass spectrometry. NMR spectroscopy was carried out using Varian Gemini 2000 spectrometer in deuterated chloroform.

### Synthesis of Fmoc-Lys(Nosyl)-OH (*N*-α-(9-Fluorenylmethyloxycarbonyl)-*N*-ε-(2-nitrobenzenesulfonyl)-L-lysine)

The solution of L-lysine hydrochloride (20 mmol) and NaHCO_3_ (20 mmol) in 20 mL of H_2_O was combined with CuSO_4_·5H_2_O (10 mmol) solution in 40 mL of H_2_O. The vigorously stirred purple solution was cooled in an ice bath and a solution of 2-nitrobenzenesulfonyl chloride (30 mmol) in acetone (60 mL) was added. Next solid NaHCO_3_ (75 mmol) was added in portions over 1 hour. The stirred reaction mixture was left in a melting ice bath overnight. The blue precipitate was filtered, subsequently washed with H_2_O, ethanol and diethyl ether (Et_2_O). After air drying yield of the complex was 87%. To the copper complex of ε-nosyl lysine (5 mmol) a solution of ethylenediaminetetraacetic acid (EDTA) disodium salt (6.5 mmol) in 40 ml of H_2_O was added. This suspension was stirred and heated at 70–80 °C until no blue complex was left and then cooled to room temperature. Afterwards, solid NaHCO_3_ (10.5 mmol) was added to the formed suspension of ε-nosyl lysine and followed by solution of Fmoc-*N*-hydoxysuccinimide ester (Fmoc-OSu) (10.5 mmol) in 30 mL of acetone. The mixture was stirred vigorously overnight, diluted with 250 mL of 1% solution of NaHCO_3_ and extracted with Et_2_O (3 × 100 mL). Ether washings were back extracted with diluted NaHCO_3_ solution and discarded. Combined aqeous phases were acidified with 10% HCl and extracted with dichloromethane (DCM) (3 × 50 mL). Combined organic phases were washed with water and dried over Na_2_SO_4_. Solvent was evaporated to afford target compound as white foam. Yield: 91%.

^1^H NMR (400 MHz, CDCl_3_) δ ppm: 8.15 – 8.06 (m, 1H), 7.85 – 7.78 (m, 1H), 7.75 (d, *J* = 7.5 Hz, 2H), 7.71 – 7.63 (m, 2H), 7.59 (d, *J* = 7.3 Hz, 2H), 7.38 (t, *J* = 7.4 Hz, 2H), 7.29 (t, *J* = 7.4 Hz, 2H), 5.48 (t, *J* = 5.8 Hz, 1H), 5.41 (d, *J* = 7.8 Hz, 1H), 4.48 – 4.29 (m, 3H), 4.21 (t, *J* = 6.6 Hz, 1H), 3.16 – 3.0 (m, 2H), 1.94 – 1.34 (m, 6H). ^13^C NMR (100 MHz, CDCl_3_) δ ppm: 176.0, 156.1, 148.0, 143.8, 143.7, 141.3, 133.5, 132.7, 131.0, 127.7, 127.1, 125.3, 125.1, 120.0, 67.2, 53.3, 47.1, 43.3, 31.6, 28.9, 21.9.

### Synthesis of carboxymethyl dithiomyristoate

Carboxymethyl dithioester was prepared in accordance to Leon *et al.*^68^. To the solution of myristic acid (4 mmol), HBTU (4 mmol) and *N*,*N*-diisopropylethylamine (DIPEA) (8 mmol) in DCM (30 mL) was added piperidine (4.2 mmol). After 4 hours reaction mixture was diluted with water and extracted with DCM. Extracts were washed with diluted HCL, diluted NaHCO_3_ and with water, dried over Na_2_SO_4_ and DCM was evaporated. To the residue toluene (10 mL) was added followed by Lawesson’s reagent (2 mmol). Reaction was heated at 106 °C for 3.5 hours. Solvent was evaporated and the residue flash-chromatographed (silica gel, ethyl acetate (EtOAc)/petr.ether 1:9) (Yield: 74%).

The solution of *N*-thiomyristoyl piperidine (3 mmol) and bromoacetic acid (3.2 mmol) in 7 mL of dried DMF was left at room temperature overnight. The solution was saturated with dry H_2_S for 30 min and DMF was evaporated in vacuo. Flash-chromatography of the residue (silica gel, DCM/AcOH 10:0.05) afforded the pure product as a yellow solid (Yield: 25%).

### Synthesis of methyl 3-[(methylthio)thiocarbonyl]propanoate

A solution of succinic anhydride (50 mmol) and piperidine (50 mmol) in EtOAc (10 mL) was refluxed for 10 min. On the next day, precipitated product was filtered, washed with EtOAc and air dried (Yield: 90%).

*N,N*-pentamethylenesuccinamic acid (10 mmol) was refluxed in anhydrous MeOH (15 mL) containing 2 drops of H_2_SO_4_ for 4 hours. Solvent was evaporated and residual oil dissolved in EtOAc, washed with NaHCO_3_ solution, water and dried over Na_2_SO_4_. Evaporation of EtOAc afforded product as a colorless oil (Yield: 84%).

Methyl *N*,*N*-pentamethylenesuccinamate (8.4 mmol) and Lawesson’s reagent (5.1 mmol) were refluxed in tetrahydrofuran (THF) (10 mL) for 1 hour. THF was evaporated in vacuum and the residue was flash-chromatographed (silica gel, EtOAc/petr.ether 1:5). Yield of slightly yellowish oil 86%.

To a solution of Methyl 3-(*N*,*N*-pentamethylenethiocarbamoyl)propanoate (2 mmol) in anhydrous THF (8 mL) MeI (10 mmol)was added. The reaction was conducted for 48 h in darkness Yellow-colored THF was decantated, the crystals were briefly washed with dry THF and dissolved in dried DMF (3 mL). Dried H_2_S was bubbled into solution for 2 h and mixture was left at 0 °C for 24 h. After addition of H_2_O (100 mL), product was extracted with EtOAc, washed several times with water, brine and dried over Na_2_SO_4_. Evaporation of EtOAc in vacuo gave the crude product as a yellow oil (Yield: 81.5%).

### Synthesis of TNFα peptide derivatives

The peptide Ac-EALPKK(NS)XGG-NH_2_ (X = T, Y(NO_2_) or Mcm) was synthesized by standard manual solid-phase-peptide synthesis using Fmoc-protected amino acid derivatives. Rink amide MBHA resin was treated with *N*,*N*-dimethylformamide (DMF) at room temperature (RT) for 10 min. The Fmoc-protecting group was removed with 20% piperidine in DMF (2 × 10 min). After washing with DMF (5 × 5 min) the resin was incubated with 4 eq of amino acid derivative, 4 eq HBTU and 8 eq of *N*,*N*-diisopropylethylamine (DIPEA) in DMF at RT (60 min). The *N*-terminus was modified with 4 eq acetic anhydride and 8 eq DIPEA in DCM (60 min). Nosyl-group was cleaved using 5 eq 1,8-Diazabicyclo[5.4.0]undec-7-en (DBU) and 5 eq thiophenol in DMF (2 × 90 min). Afterwards the resin was washed with DMF. Free lysine side chain was modified on-resin with HBTU (4 eq.), DIPEA (8 eq.) in DCM/DMF mixure (1:1) and myristic acid (**1a**, **2a**), 6-(Fmoc-amino)-caproic acid and *N*-Boc-anthranilic acid (**4**, **5**) or 8-(Fmoc-amino)-octanoic acid and *N*-Boc-anthranilic acid (**4a**). For **3** free lysine residue was acylated with 11-azidoundecanoic acid^69^ according to the method used for peptides **1a** and **1b**. The resin was treated with a solution of triphenylphosphine (5 eq) in tetrahydrofuran (THF)/H_2_O (95:5) for several days (small portions of resin were taken for the test cleavage and MS-analysis). After washing *N*-Boc-anthranilic acid was coupled by the standard method (see peptides **1a** and **2b**). **6** and **7** were prepared like peptide **3** with (4-*N*,*N*-dimethylamino-1,8-naphthalimid)-acetic acid^64^ (**6**)or 2-amino-5-nitrobenzoic acid (**7**) instead of *N*-Boc-anthranilic acid. For **8** the resin bound peptide was incubated with the solution of carboxymethyl dithiomyristoate (3 eq) and DIPEA (3 eq) in DMF for 3 h and cleaved as described in general procedure. For **9** an an ice-cooling prepared solution of 5 eq of triphenylphosphine, 5 eq of diethyl azodicarboxylate (DIAD) and 10 eq of dried MeOH in dried DCM was added to the resin-bound fully protected peptide with the ε-Nosyl-protected lysine (Mitsunoby reaction). After one hour of incubation the resin was washed 5 times with DMF. Nosyl-group was removed as described above and resin was treated with a solution of carboxymethyl dithiomyristoate (3 eq) and DIPEA (3 eq) in DMF for 3 h.

Peptides **10** and **11** are based on a CPS1-peptide (Bz-GVLKEYGV-NH_2_). To a DMF solution of the CPS1 peptide, ethyl dithioacetate (1.2 eq) and triethylamine (5 eq) (**10**) or methyl 3-[(methylthio)thiocarbonyl]propanoate (1.1 eq) and triethylamine (5 eq) (**11**) were added. Reaction mixture was stirred for 3–5 h. For **11** 1 M NaOH (6 eq) was added and stirring continued for another 2 h.

The cyclic peptide inhibitor S2iL5 was synthesized by standard Fmoc-based solid phase peptide synthesis as described by Yamagata *et al.*^60^.

All 3-nitrotyrosine containing peptides (**2a**, **2b**, **3**, **4, 4a**, **5** and **6**) require an additional piperidine treatment step (20% piperidine in DMF, 2 × 10 min) to remove acyl-group from 3-nitrotyrosine before cleavage.

The resin was washed with DCM (5 × 4 min), methanol (3 × 4 min) and DCM again and treated with TFA/H_2_O (98:2) (2 × 60 min). Combined TFA solutions were evaporated in vacuum and re-dissolved in ACN/H_2_O solution (50:50). HPLC purification and subsequent lyophilization yielded pure peptides.

### Expression and purification of human sirtuins

Sirt1, Sirt2, Sirt3, Sirt5 and Sirt6 were expressed and purified as described before^26^,^30^,^70^,^71^.

To obtain the expression plasmid of human (His)_6_-SUMO-Sirt4(29–314), the respective DNA fragment was PCR-amplified using gene-specific primers from the plasmid pET101-Sirt4, which carries the Sirt4 gene, and cloned into the BsaI, XbaI sites of pE-SUMO yielding the plasmid pE-SUMO-Sirt4(29–314).

The protein was overexpressed in *E. coli* BL21 (DE3) cells at 18 °C. The purification of the protein was performed using affinity chromatography on Ni-NTA resin in 10 mM Tris-HCl, pH 7.5, 0.5 M NaCl. The matrix-bound (His)_6_-SUMO-Sirt4(29–314) was eluted by imidazole in the buffer and further purified by gel filtration in 10 mM HEPES, pH 7.8, 150 mM KCl, 1.5 mM MgCl_2_, and stored at −20 °C for use.

pQE-80L (Qiagen, Valencia, Ca) His-tagged Sirt6 (1–355) was transformed into the competent *E. coli* strain, BL21 DE3 and overexpressed at room temperature. For purification a nickel resin affinity chromatography was used in 50 mM NaPO_4_ pH 7.2, 250 mM NaCl, 5 mM imidazol, 1mM BME and eluted by 250 mM imidazol in the buffer. Furthermore, Sirt6 was purified secondarily via a HiTrap SP-Sepharose Fast Flow column (GE Healthcare) using a linear gradient from 50–750 mM NaCl in 50 mM NaPO_4_ pH 7.2, 1 mM BME. Afterwards fractions containing purified Sirt6 were pooled, concentrated and dialyzed into 50 mM Tris, pH 8.0 (4 °C), 150 mM NaCl, 100 μM TCEP and 5% (w/v) glycerol and stored at −70 °C.

### HPLC based activity assay

For the determination of kinetic constants for all sirtuin mediated reactions solutions containing 20 mM TRIS/HCl pH 7.8, 150 mM NaCl, 5 mM MgCl_2_ (assay-buffer), 500 μM NAD^+^ and varying substrate concentrations (0.5–100 μM) were used. Deacylation was started by adding human sirtuin to reach a final concentration of 0.01–0.5 μM. Enzyme-catalyzed reaction was stopped using TFA (1% final concentration) after 1 min to 180 min of incubation at 37 °C depending on substrate reactivity. The cleavage rate of the different TNFα peptide derivatives was analyzed using analytical reversed phase HPLC. 40 to 80 μl of compounds or reaction solutions were injected and separated using a linear gradient from 5% to 95% solvent B within 6min. The product and substrate peaks were quantified using absorbance at 220 nm or 365 nm (absorption of 3-Nitrotyrosyl moiety). The peak areas were integrated and converted to initial velocity rates calculated from the ratio of product area to total peak area. Linear regression of conversions plotted against time yielded reaction rates in μM/min (relative conversion below 20% of substrate). Non-linear regression according to Michaelis-Menten of the reaction rates at different substrate concentrations yielded K_M_- and k_cat_-values using the program SigmaPlot 8 (Systat Software, San Jose, USA). All measurements were done in duplicates.

### Continuous fluorescence assay

The fluorescence measurements were performed on a Hitachi F-4500 fluorescence spectrophotometer (Tokyo, Japan) at λ_Ex_ = 310 nm and λ_Em_ = 405 nm (slit_Ex_ = 5 nm, slit_Em_ = 2.5 nm, PMT = 700 V for **3**, **4** and **5** as well as slit_Ex_ = 10 nm, slit_Em_ = 10 nm, PMT = 950 V for **4a**). Each reaction mixture contained assay-buffer, 0.5 mM NAD^+^ and various peptide concentrations (0.1–100 μM) and was preincubated for 5 minutes at 37 °C. The reaction was started by adding human sirtuin (0.1–0.5 μM) and observed for 5–10 minutes. Product formation could be monitored by increase of relative fluorescence. This signal was converted into product concentration via calibration lines. The slope of the linear regression of product formation against time yielded the reaction velocity rates in μM/s. K_M_ and k_cat_ were obtained by non-linear regression according to Michaelis-Menten. All measurements were done in duplicates. For determination of reaction velocity rates in μM/s calibration lines were necessary. Therefore a reaction mixture was prepared, containing assay-buffer, 2 μM Sirt2, 500 μM NAD^+^ and 100 μM of **3**, **4, 4a** or **5** was incubated overnight at 37 °C. The reaction mixture was analyzed with HPLC, to control if the entire peptide substrate was turned to product. Additionally the mixture was diluted (0.1–25 μM) and measured with Hitachi F-4500 fluorescence spectrophotometer at the same conditions as described above.

The microtiter plate fluorescence measurements were performed on a Tecan Infinite M200 microplate reader (Maennedorf, Switzerland) at λ_Ex_ = 320 nm and λ_Em_ = 408 nm (lag time 9 μs, integration time 20 μM, gain 160, 170 or 182). The reactions (total volume 100 μl) were measured in black low-binding 96- well microtiter plates (NUNC). Assay-buffer, 500 μM NAD^+^ and 0.07–200 μM peptide substrate were pre-incubated at 37 °C for 5 min. The reaction was started by adding human sirtuin (0.01–0.5 μM). The signals were converted into product concentration via calibration lines and the resulting data were evaluated as described above (single fluorescence measurement). The determination for the kinetic constants of NAD^+^ was performed in the same way, except that the peptide concentration was fixed (5, 25 or 200 μM) and the NAD^+^ concentration was varied (10–1500 μM). All measurements were done in duplicates. The reaction mixture for the calibration lines was prepared as described for the single fluorescence measurements. After complete turnover of peptide substrate **3**, the solution was diluted (0.2–20 μM) and measured on Tecan infinite M200 microplate reader at λ_Ex_ = 320 nm and λ_Em_ = 408 nm (lag time 9 μs, integration time 20 μM, gain 182 (G182), 170 (G170) and 160 (G160)) K_i_ values of the inhibitors were determined by recording k_cat_ and K_M_ values for **3** in the presence of varying inhibitor concentrations (0.01–600 μM). The resulting plots were analyzed by a competitive inhibition and non-competitive inhibition model using the program Sigma Plot 8. The linear regression of the apparent K_M_-values against the corresponding inhibitor concentration yielded the inhibitor constant K_i_ for competitive inhibition. The K_i_ for non-competitive inhibition was determined by linear regression of 1/apparent V_max_ against the corresponding inhibitor concentration. The negative K_i_ value can be determined as intersection with the X-axis from these plots.

### Photo-induced change of *cis* content of thioxo peptides

Excitation experiments of thioxo peptides were done in a cuvette at 254 nm under stirring with a UV-lamp (UV handheld lamp, Carl Roth). For irradiation a distance of 5 cm between cuvette and UV-lamp was chosen. UV-spectra were recorded between 230 and 325 nm using a spectrophotometer (Specord M500).

For determination of temperature dependent *cis*/*trans* isomerization a 50 μM solution of thioxo peptide was incubated for 10 min at different temperatures (10–70 °C). UV-spectra were recorded at ground state (GS) and after irradiation at 254 nm (irradiation time 45 s to 5 min) at photostationary state (PSS). Several UV-spectra over time were recorded to determine rates of *cis*/*trans* isomerization. Using a differential spectrum (UV spectrum GS – UV spectrum PSS) activation parameter and isomerization velocity could be examined.

The *cis*/*trans* content of a 50 μM thioxo peptide solution was changed by 5 min irradiation at 254 nm and the resulting solution was analysed by HPLC. Additionally, several solvents were tested to enhance *cis* content. As solvents H_2_O, acetic acid, TFA, trifluoro ethanol (TFE), 0.5 M LiCl in H_2_O/ethanol (EtOH)/TFE, methanol (MeOH), formic acid, *N*-methyl pyrolidon (NMP), DMF, Dimethylsulfoxid (DMSO) and tetrahydrofuran (THF) were chosen. *Cis* content was determined via HPLC of a 500 μM solution of **9**.

For the separation of isomers, 5–6 mg of **9** were dissolved in 50% ACN and equilibrated overnight. For better separation HPLC-solvents were cooled down to 4 °C and a linear gradient of 45% solvent B to 55% solvent B in 70 min was used. Eluted fractions were immediately frozen in liquid nitrogen. HPLC-based determination of *cis* content was done directly after preparative separation.

The examination of the isomer specific inhibition of **8**, **9**, **10** and **11** was examined via HPLC using reaction solutions composed of 500 μM NAD^+^, 30 μM peptide, 0.5 μM sirtuin and 0.5–40 μM inhibitor in GS, PSS or GS* in assay buffer. After 30 min incubation at 20 °C reaction was stopped using 10% TFA solution. Inhibitor solutions were irradiated at 254 nm for 5 min. Separated isomers were applied in concentrations from 1 to 10 μM. The influence of *cis* content on sirtuin inhibition using fluorescence spectrometer was determined with 500 μM NAD^+^, 5 μM **3**, 0.1 μM Sirt2 and 0.1 μM **9**. Reactions were done at 20 °C with **9** in GS and PSS (after 5 min irradiation at 254 nm). **9** in PSS was applied immediately after irradiation (transfer time ~5 s) and started directly by adding sirtuin. Reactions were measured within 1 min to avoid re-isomerization.

### Z’ factor analysis

The Z′ factor is a dimensionless, simple statistic parameter for high-throughput screening assays^72^. It is defines as the ratio of separation band to signal dynamic range of the assay and used the signal variation at the two extremes of the activity range (0 and 100% activity).




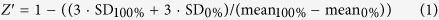




## Additional Information

**How to cite this article**: Schuster, S. *et al.* A continuous sirtuin activity assay without any coupling to enzymatic or chemical reactions. *Sci. Rep.* **6**, 22643; doi: 10.1038/srep22643 (2016).

## Supplementary Material

Supplementary Information

## Figures and Tables

**Table 1:** Kinetic constants for **3**, **4**, **4a** and **5** and Sirt2.

Substrate	K_M_ [μM]	10^−3^xk_cat_ [s^−1^]	k_cat_/K_M_ [M^−1^s^−1^]	Distance (No. of bonds)
**4**	17.7 ± 1.5	4.0 ± 0.1	224^a^	6
**4a**	1.2 ± 0.1	45.4 ± 1.8	38 600^b^	8
**3**	0.1 ± 0.02	23.8 ± 0.8	176 000^a^	11
**5**	15.3 ± 2.7	2.4 ± 0.1	156^b^	13

^a^measured using fluorescence spectrophotometer.

^b^measured using MTP fluorescence reader (see supporting information for details). Data are presented as mean ± s.d. (n = 2).

**Table 2:** Kinetic constants for 3 and Sirt1-6.

Enzyme	K_M_ [μM]	10^−3^xk_cat_ [s^−1^]	k_cat_/K_M_ [M^−1^s^−1^]	c (Sirt) [nM]
Sirt1	0.7 ± 0.08	2.1 ± 0.1	287^a^	500
Sirt2	0.12 ± 0.02	23.8 ± 0.8	176 000^a^	10
Sirt3	3.3 ± 0.4	9.1 ± 0.4	2 800^a^	100
Sirt4	49.5 ± 7.5	0.4 ± 0.02	7^a^	1000
Sirt5	46.1 ± 7.2	3.2 ± 0.2	69^b^	500
Sirt6	23.5 ± 4.9	0.9 ± 0.1	39^a^	500

^a^measured using fluorescence spectrophotometer.

^b^measured using MTP fluorescence reader (see supporting information for details). Data are presented as mean ± s.d. (n = 2).

**Table 3:** K_i_-values for different inhibitors.

inhibitor	Enzyme	K_i_ (3) [μM]	K_i_ (NAD^+^) [μM]
NAM	Sirt3	93.0 ± 8.5	45.0 ± 14.2
	Sirt6	451.0 ± 60.7	415.0 ± 45.1
Ex-527	Sirt6	100.0 ± 11.0	n.d.
Quercetin	Sirt6	21.0 ± 3.4	n.d.
S2iL5	Sirt2	0.6 ± 0.2	n.d.
**8**	Sirt2	0.08 ± 0.02	n.d.
	Sirt3	0.1 ± 0.02	n.d.
	Sirt6	0.4 ± 0.1	n.d.
	Sirt6^a^	1.1 ± 0.1^e^	n.d.
	Sirt6^b^	1.9 ± 0.2^e^	n.d.
**9**	Sirt6^c^	0.6 ± 0.1^e^	n.d.
	Sirt6^d^	1.7 ± 0.5^e^	n.d.
**10**	Sirt2	0.3 ± 0.1	n.d.
**11**	Sirt2	50.0 ± 9.9	n.d.

^a^2.5% *cis* isomer.

^b^25% *cis* isomer.

^c^72.4% *cis* isomer.

^d^29.7% *cis* isomer.

^e^IC_50_ value, n.d. not determined.

## References

[b1] Choudhary C., Weinert B. T., Nishida Y., Verdin E. & Mann M. The growing landscape of lysine acetylation links metabolism and cell signalling. Nat Rev Mol Cell Biol 15, 536–550 (2014). 25053359 10.1038/nrm3841

[b2] Zhao S. *et al.* Regulation of cellular metabolism by protein lysine acetylation. Science 327, 1000–1004 (2010). 20167786 10.1126/science.1179689 PMC3232675

[b3] Chen Y. *et al.* Lysine propionylation and butyrylation are novel post-translational modifications in histones. Mol Cell Proteomics 6, 812–819 (2007). 17267393 10.1074/mcp.M700021-MCP200 PMC2911958

[b4] Garrity J., Gardner J. G., Hawse W., Wolberger C. & Escalante-Semerena J. C. N-lysine propionylation controls the activity of propionyl-CoA synthetase. J Biol Chem 282, 30239–30245 (2007). 17684016 10.1074/jbc.M704409200

[b5] Zhang Z. *et al.* Identification of lysine succinylation as a new post-translational modification. Nat Chem Biol 7, 58–63 (2011). 21151122 10.1038/nchembio.495 PMC3065206

[b6] Xie Z. *et al.* Lysine succinylation and lysine malonylation in histones. Mol Cell Proteomics 11, 100–107 (2012). 22389435 10.1074/mcp.M111.015875 PMC3418837

[b7] Peng C. *et al.* The first identification of lysine malonylation substrates and its regulatory enzyme. Mol Cell Proteomics 10, M111 012658 (2011). 10.1074/mcp.M111.012658 PMC3237090 21908771

[b8] Tan M. *et al.* Lysine glutarylation is a protein posttranslational modification regulated by SIRT5. Cell Metab 19, 605–617 (2014). 24703693 10.1016/j.cmet.2014.03.014 PMC4108075

[b9] Tan M. *et al.* Identification of 67 histone marks and histone lysine crotonylation as a new type of histone modification. Cell 146, 1016–1028 (2011). 21925322 10.1016/j.cell.2011.08.008 PMC3176443

[b10] Dai L. *et al.* Lysine 2-hydroxyisobutyrylation is a widely distributed active histone mark. Nat Chem Biol 10, 365–370 (2014). 24681537 10.1038/nchembio.1497

[b11] Moellering R. E. & Cravatt B. F. Functional lysine modification by an intrinsically reactive primary glycolytic metabolite. Science 341, 549–553 (2013). 23908237 10.1126/science.1238327 PMC4005992

[b12] Stevenson F. T., Bursten S. L., Fanton C., Locksley R. M. & Lovett D. H. The 31-kDa precursor of interleukin 1 alpha is myristoylated on specific lysines within the 16-kDa N-terminal propiece. Proc Natl Acad Sci USA 90, 7245–7249 (1993). 8346241 10.1073/pnas.90.15.7245 PMC47113

[b13] Simic Z., Weiwad M., Schierhorn A., Steegborn C. & Schutkowski M. The epsilon-Amino Group of Protein Lysine Residues Is Highly Susceptible to Nonenzymatic Acylation by Several Physiological Acyl-CoA Thioesters. Chembiochem 16, 2337–2347 (2015). 26382620 10.1002/cbic.201500364

[b14] Wagner G. R. & Payne R. M. Widespread and enzyme-independent Nepsilon-acetylation and Nepsilon-succinylation of proteins in the chemical conditions of the mitochondrial matrix. J Biol Chem 288, 29036–29045 (2013). 23946487 10.1074/jbc.M113.486753 PMC3790002

[b15] Weinert B. T. *et al.* Acetyl-phosphate is a critical determinant of lysine acetylation in E. coli. Mol Cell 51, 265–272 (2013). 23830618 10.1016/j.molcel.2013.06.003

[b16] Kuhn M. L. *et al.* Structural, kinetic and proteomic characterization of acetyl phosphate-dependent bacterial protein acetylation. PLoS One 9, e94816 (2014). 24756028 10.1371/journal.pone.0094816 PMC3995681

[b17] Avalos J. L., Boeke J. D. & Wolberger C. Structural basis for the mechanism and regulation of Sir2 enzymes. Mol Cell 13, 639–648 (2004). 15023335 10.1016/s1097-2765(04)00082-6

[b18] Du J. *et al.* Sirt5 is a NAD-dependent protein lysine demalonylase and desuccinylase. Science 334, 806–809 (2011). 22076378 10.1126/science.1207861 PMC3217313

[b19] Park J. *et al.* SIRT5-mediated lysine desuccinylation impacts diverse metabolic pathways. Mol Cell 50, 919–930 (2013). 23806337 10.1016/j.molcel.2013.06.001 PMC3769971

[b20] Feldman J. L., Baeza J. & Denu J. M. Activation of the protein deacetylase SIRT6 by long-chain fatty acids and widespread deacylation by mammalian sirtuins. J Biol Chem 288, 31350–31356 (2013). 24052263 10.1074/jbc.C113.511261 PMC3829447

[b21] Jiang H. *et al.* SIRT6 regulates TNF-alpha secretion through hydrolysis of long-chain fatty acyl lysine. Nature 496, 110–113 (2013). 23552949 10.1038/nature12038 PMC3635073

[b22] Mathias R. A. *et al.* Sirtuin 4 is a lipoamidase regulating pyruvate dehydrogenase complex activity. Cell 159, 1615–1625 (2014). 25525879 10.1016/j.cell.2014.11.046 PMC4344121

[b23] Bao X. *et al.* Identification of ‘erasers’ for lysine crotonylated histone marks using a chemical proteomics approach. Elife 3, e02999 (2014). 10.7554/eLife.02999 PMC4358366 25369635

[b24] Teng Y. B. *et al.* Efficient demyristoylase activity of SIRT2 revealed by kinetic and structural studies. Sci Rep 5, 8529 (2015). 25704306 10.1038/srep08529 PMC4894398

[b25] He B., Hu J., Zhang X. & Lin H. Thiomyristoyl peptides as cell-permeable Sirt6 inhibitors. Org Biomol Chem 12, 7498–7502 (2014). 25163004 10.1039/c4ob00860j PMC4161628

[b26] Moniot S., Schutkowski M. & Steegborn C. Crystal structure analysis of human Sirt2 and its ADP-ribose complex. J Struct Biol 182, 136–143 (2013). 23454361 10.1016/j.jsb.2013.02.012

[b27] Haigis M. C. & Guarente L. P. Mammalian sirtuins—emerging roles in physiology, aging, and calorie restriction. Genes Dev 20, 2913–2921 (2006). 17079682 10.1101/gad.1467506

[b28] Schutkowski M., Fischer F., Roessler C. & Steegborn C. New assays and approaches for discovery and design of Sirtuin modulators. Expert Opin Drug Discov 9, 183–199 (2014). 24382304 10.1517/17460441.2014.875526

[b29] Hubbard B. P. *et al.* Evidence for a common mechanism of SIRT1 regulation by allosteric activators. Science 339, 1216–1219 (2013). 23471411 10.1126/science.1231097 PMC3799917

[b30] Gertz M. *et al.* A molecular mechanism for direct sirtuin activation by resveratrol. PLoS One 7, e49761 (2012). 23185430 10.1371/journal.pone.0049761 PMC3504108

[b31] Li Y. *et al.* A mini-review on Sirtuin activity assays. Biochem Biophys Res Commun 3, 459–466 (2015). 10.1016/j.bbrc.2015.09.172 26456653

[b32] Fan Y. & Scriba G. K. Electrophoretically mediated microanalysis assay for sirtuin enzymes. Electrophoresis 31, 3874–3880 (2010). 21064140 10.1002/elps.201000336

[b33] Tanner K. G., Landry J., Sternglanz R. & Denu J. M. Silent information regulator 2 family of NAD- dependent histone/protein deacetylases generates a unique product, 1-O-acetyl-ADP-ribose. Proc Natl Acad Sci USA 97, 14178–14182 (2000). 11106374 10.1073/pnas.250422697 PMC18891

[b34] Roessler C. *et al.* Chemical probing of the human sirtuin 5 active site reveals its substrate acyl specificity and Peptide-based inhibitors. Angew Chem Int Ed Engl 53, 10728–10732 (2014). 25111069 10.1002/anie.201402679

[b35] Fischer F. *et al.* Sirt5 deacylation activities show differential sensitivities to nicotinamide inhibition. PLoS One 7, e45098 (2012). 23028781 10.1371/journal.pone.0045098 PMC3446968

[b36] Rye P. T., Frick L. E., Ozbal C. C. & Lamarr W. A. Advances in label-free screening approaches for studying sirtuin-mediated deacetylation. J Biomol Screen 16, 1217–1226 (2011). 21911826 10.1177/1087057111420291

[b37] Marcotte P. A. *et al.* Fluorescence assay of SIRT protein deacetylases using an acetylated peptide substrate and a secondary trypsin reaction. Anal Biochem 332, 90–99 (2004). 15301953 10.1016/j.ab.2004.05.039

[b38] Smith B. C., Hallows W. C. & Denu J. M. A continuous microplate assay for sirtuins and nicotinamide-producing enzymes. Anal Biochem 394, 101–109 (2009). 19615966 10.1016/j.ab.2009.07.019 PMC2752052

[b39] Wolfson N. A., Pitcairn C. A., Sullivan E. D., Joseph C. G. & Fierke C. A. An enzyme-coupled assay measuring acetate production for profiling histone deacetylase specificity. Anal Biochem 456, 61–69 (2014). 24674948 10.1016/j.ab.2014.03.012 PMC4470474

[b40] Wegener D., Wirsching F., Riester D. & Schwienhorst A. A fluorogenic histone deacetylase assay well suited for high-throughput activity screening. Chem Biol 10, 61–68 (2003). 12573699 10.1016/s1074-5521(02)00305-8

[b41] Baba R., Hori Y., Mizukami S. & Kikuchi K. Development of a fluorogenic probe with a transesterification switch for detection of histone deacetylase activity. J Am Chem Soc 134, 14310–14313 (2012). 22917182 10.1021/ja306045j

[b42] Minoshima M., Matsumoto T. & Kikuchi K. Development of a fluorogenic probe based on a DNA staining dye for continuous monitoring of the histone deacetylase reaction. Anal Chem 86, 7925–7930 (2014). 25004201 10.1021/ac501881s

[b43] Han Y. *et al.* Time-Resolved Luminescence Biosensor for Continuous Activity Detection of Protein Acetylation-Related Enzymes Based on DNA-Sensitized Terbium(III) Probes. Anal Chem 87, 9179–9185 (2015). 26307596 10.1021/acs.analchem.5b01338

[b44] Dhara K., Hori Y., Baba R. & Kikuchi K. A fluorescent probe for detection of histone deacetylase activity based on aggregation-induced emission. Chem Commun (Camb) 48, 11534–11536 (2012). 23093210 10.1039/c2cc36591j

[b45] Wang Y., Chen Y., Wang H., Cheng Y. & Zhao X. Specific Turn-On Fluorescent Probe with Aggregation-Induced Emission Characteristics for SIRT1 Modulator Screening and Living-Cell Imaging. Anal Chem 10, 5046–5049 (2015). 10.1021/acs.analchem.5b01069 25903518

[b46] Liu Y., Gerber R., Wu J., Tsuruda T. & McCarter J. D. High-throughput assays for sirtuin enzymes: a microfluidic mobility shift assay and a bioluminescence assay. Anal Biochem 378, 53–59 (2008). 18358225 10.1016/j.ab.2008.02.018

[b47] Suenkel B., Fischer F. & Steegborn C. Inhibition of the human deacylase Sirtuin 5 by the indole GW5074. Bioorg Med Chem Lett 23, 143–146 (2013). 23195732 10.1016/j.bmcl.2012.10.136

[b48] Hubbard B. P. & Sinclair D. A. Measurement of sirtuin enzyme activity using a substrate-agnostic fluorometric nicotinamide assay. Methods Mol Biol 1077, 167–177 (2013). 24014406 10.1007/978-1-62703-637-5_11 PMC4727966

[b49] Howitz K. T. *et al.* Small molecule activators of sirtuins extend Saccharomyces cerevisiae lifespan. Nature 425, 191–196 (2003). 12939617 10.1038/nature01960

[b50] Madsen A. S. & Olsen C. A. Substrates for efficient fluorometric screening employing the NAD-dependent sirtuin 5 lysine deacylase (KDAC) enzyme. J Med Chem 55, 5582–5590 (2012). 22583019 10.1021/jm300526r

[b51] Hu J., He B., Bhargava S. & Lin H. A fluorogenic assay for screening Sirt6 modulators. Org Biomol Chem 11, 5213–5216 (2013). 23839075 10.1039/c3ob41138a PMC3756594

[b52] Kaeberlein M. *et al.* Substrate-specific activation of sirtuins by resveratrol. J Biol Chem 280, 17038–17045 (2005). 15684413 10.1074/jbc.M500655200

[b53] Pacholec M. *et al.* SRT1720, SRT2183, SRT1460, and resveratrol are not direct activators of SIRT1. J Biol Chem 285, 8340–8351 (2010). 20061378 10.1074/jbc.M109.088682 PMC2832984

[b54] Li Y., Huang W., You L., Xie T. & He B. A FRET-based assay for screening SIRT5 specific modulators. Bioorg Med Chem Lett 25, 1671–1674 (2015). 25818461 10.1016/j.bmcl.2015.03.018

[b55] Li Y. *et al.* A FRET-based assay for screening SIRT6 modulators. Eur J Med Chem 96, 245–249 (2015). 25884115 10.1016/j.ejmech.2015.04.008

[b56] Roessler C., Tuting C., Meleshin M., Steegborn C. & Schutkowski M. A Novel Continuous Assay for the Deacylase Sirtuin 5 and Other Deacetylases. J Med Chem 58, 7217–7223 (2015). 26308971 10.1021/acs.jmedchem.5b00293

[b57] Feldman J. L. *et al.* Kinetic and Structural Basis for Acyl-Group Selectivity and NAD Dependence in Sirtuin-Catalyzed Deacylation. Biochemistry 19, 3037–3050 (2015). 10.1021/acs.biochem.5b00150 PMC4470489 25897714

[b58] Kokkonen P. *et al.* Studying SIRT6 regulation using H3K56 based substrate and small molecules. Eur J Pharm Sci 63, 71–76 (2014). 25004411 10.1016/j.ejps.2014.06.015

[b59] Smith B. C. & Denu J. M. Acetyl-lysine analog peptides as mechanistic probes of protein deacetylases. J Biol Chem 282, 37256–37265 (2007). 17951578 10.1074/jbc.M707878200

[b60] Yamagata K. *et al.* Structural basis for potent inhibition of SIRT2 deacetylase by a macrocyclic peptide inducing dynamic structural change. Structure 22, 345–352 (2014). 24389023 10.1016/j.str.2013.12.001

[b61] Fatkins D. G., Monnot A. D. & Zheng W. Nepsilon-thioacetyl-lysine: a multi-facet functional probe for enzymatic protein lysine Nepsilon-deacetylation. Bioorg Med Chem Lett 16, 3651–3656 (2006). 16697640 10.1016/j.bmcl.2006.04.075

[b62] Smith B. C. & Denu J. M. Mechanism-based inhibition of Sir2 deacetylases by thioacetyl-lysine peptide. Biochemistry 46, 14478–14486 (2007). 18027980 10.1021/bi7013294

[b63] Fatkins D. G. & Zheng W. Substituting N(epsilon)-thioacetyl-lysine for N(epsilon)-acetyl-lysine in peptide substrates as a general approach to inhibiting human NAD(+)-dependent protein deacetylases. Int J Mol Sci 9, 1–11 (2008). 19325715 10.3390/ijms9010001 PMC2635597

[b64] Loving G. & Imperiali B. A versatile amino acid analogue of the solvatochromic fluorophore 4-N,N-dimethylamino-1,8-naphthalimide: a powerful tool for the study of dynamic protein interactions. J Am Chem Soc 130, 13630–13638 (2008). 18808123 10.1021/ja804754y PMC2647014

[b65] Zhao J., Micheau J. C., Vargas C. & Schiene-Fischer C. cis/trans photoisomerization of secondary thiopeptide bonds. Chemistry 10, 6093–6101 (2004). 15515071 10.1002/chem.200400400

[b66] Wildemann D. *et al.* A nearly isosteric photosensitive amide-backbone substitution allows enzyme activity switching in ribonuclease s. J Am Chem Soc 129, 4910–4918 (2007). 17397159 10.1021/ja069048o

[b67] Frank R., Jakob M., Thunecke F., Fischer G. & Schutkowski M. Thioxylation as One-Atom-Substitution Generates a Photoswitchable Element within the Peptide Backbone We thank Dr. Peter Bayer for the NMR investigations and Dirk Wildemann for his assistance with the peptide syntheses. This work was supported by the Deutsche Forschungsgemeinschaft, the Fond der Chemischen Industrie, the Boehringer-Ingelheim-Stiftung, and the Land Sachsen-Anhalt. Angew Chem Int Ed Engl 39, 1120–1122 (2000). 10760939

[b68] Leon N. H. New compounds: synthesis of carboxymethyl carbodithioates. J Pharm Sci 65, 146–148 (1976). 1255422 10.1002/jps.2600650139

[b69] Gubbens J. *et al.* Photocrosslinking and click chemistry enable the specific detection of proteins interacting with phospholipids at the membrane interface. Chem Biol 16, 3–14 (2009). 19171301 10.1016/j.chembiol.2008.11.009

[b70] Schlicker C., Boanca G., Lakshminarasimhan M. & Steegborn C. Structure-based development of novel sirtuin inhibitors. Aging (Albany NY) 3, 852–872 (2011). 21937767 10.18632/aging.100388 PMC3227451

[b71] Ryu D. *et al.* A SIRT7-dependent acetylation switch of GABPbeta1 controls mitochondrial function. Cell Metab 20, 856–869 (2014). 25200183 10.1016/j.cmet.2014.08.001

[b72] Zhang J. H., Chung T. D. & Oldenburg K. R. A Simple Statistical Parameter for Use in Evaluation and Validation of High Throughput Screening Assays. J Biomol Screen 4, 67–73 (1999). 10838414 10.1177/108705719900400206

