# Supplementary material for: A continuous sirtuin activity assay without any coupling to enzymatic or chemical reactions
Source: Sci Rep. 2016 Mar 4;6:22643. doi: 10.1038/srep22643 (PMC4778124; doi:10.1038/srep22643)
Supplement: Supplementary Information [file srep22643-s1.docx]

A continuoust sirtuin activity assay without any coupling to enzymatic or chemical reactions

Sabine Schuster^1*^, Claudia Roessler^1^*, Marat Meleshin^1^, Philipp Zimmermann^1,2^, Zeljko Simic^1^, Christian Kambach^4^, Cordelia Schiene-Fischer^3^, Clemens Steegborn^4^, Michael O. Hottiger^5^, Mike Schutkowski^1^

^1^ Department of Enzymology, Institute of Biochemistry and Biotechnology, Martin-Luther-University Halle-Wittenberg, Kurt-Mothes-Strasse 3, 0610 Halle (Saale), Germany

^2^ present address: Department of Biotechnology, Institute of Biochemistry and Biotechnology, Martin-Luther-University Halle-Wittenberg, Kurt-Mothes-Strasse 3, 0610 Halle (Saale), Germany

^3^ Department of Enzymology, joint research project gFP5, Institute of Biochemistry and Biotechnology, Martin-Luther-University Halle-Wittenberg, Kurt-Mothes-Strasse 3, 0610 Halle (Saale), Germany

^4^ Department of Biochemistry, University of Bayreuth, Universitaetsstrasse 30, 95447 Bayreuth, Germany

^5^ IVBMB, University of Zurich-Irchel, Winterthurerstrasse 190, 8057 Zurich, Switzerland

* These authors contributed equally to this work

Content

[Synthesis 3](#_Toc433049045)

[Enzymatic measurements 3](#_Toc433049046)

[HPLC based activity assay 3](#_Toc433049047)

[Product formation for different substrates 3](#_Toc433049048)

[Determination of kinetic constants 4](#_Toc433049049)

[Influence of resveratrol on sirtuin reaction 5](#_Toc433049050)

[Single Fluorescence Measurements 5](#_Toc433049051)

[Control Measurements 5](#_Toc433049052)

[Determination of kinetic constants 6](#_Toc433049053)

[Calibration Lines 7](#_Toc433049054)

[Simultaneous measurments of sirtuin activity using two substrates 8](#_Toc433049055)

[Microtiter plate fluorescence measurements 10](#_Toc433049056)

[Determination of kinetic constants 10](#_Toc433049057)

[Determination of the inhibitor constant K_i_ 11](#_Toc433049058)

[Calibration Curves 14](#_Toc433049059)

[Overview kinetic constants 14](#_Toc433049060)

[Photo-induced change of *cis* content of thioxo peptides 15](#_Toc433049061)

[Temperature dependency of *cis*/*trans* isomerization 15](#_Toc433049062)

[Determination of *cis*/*trans* content 30](#_Toc433049063)

[Separation of isomers 30](#_Toc433049064)

[Determination of isomer specific inhibition of sirtuins by thioxo peptides 31](#_Toc433049065)

[NMR spectra 33](#_Toc433049066)

[HPLC chromatograms and MS spectra of peptide derivatives 35](#_Toc433049067)

# Synthesis

**Supplementary Figure S1.** Synthesis of carboxymethyl dithiomyristoate.

**Supplementary Figure S2**. Synthesis of methyl 3-[(methylthio)thiocarbonyl]propanoate.

# Enzymatic measurements

## HPLC based activity assay

### Product formation for different substrates

**Supplementary Table S1.** Substrate conversion of different peptide substrates and sirtuins.

| Peptide | Sirt2  product in % | | Sirt3  product in% | | Sirt6  product in % | |  |
| --- | --- | --- | --- | --- | --- | --- | --- |
|  | 0.5 h | 1 h | 0.5 h | 1h | 0.5 h | 1 h | |
| **13** | 38.2 | 62.2 | 46.6 | 80.5 | 13.9 | 19.6 | |
| **16** | 30.9 | 48.1 | 42.2 | 69.5 | 16.3 | 23.5 | |

The reactions were performed with 0.1- 0.5 µM sirtuin, 500 µM NAD^+^ and 50µM **13**/**16**. All reactions were done in duplicates (n=2).

### Determination of kinetic constants

| **Sirt2**  **A** | ****  **B** |
| --- | --- |
| **Sirt3**  **A** | ****  **B** |
| **Sirt5**  **A** | ****  **B** |
| **Sirt6**  **A** | **B** |

**Supplementary Figure S3.** Kinetic characteristics of **1a** **(A)** and **3** **(B)** for different sirtuins. The reactions were performed with 0.1- 0.5 µM sirtuin, 500 µM NAD^+^ and varying concentrations of substrates (0.5-100 µM). The reactions were analyzed by HPLC. Data are presented as mean ± s.d. (n=2) and lines represent the Michaelis-Menten-plot.

### Influence of resveratrol on sirtuin reaction





**Supplementary Figure S4.** Resveratrol mediated activity of Sirt1. Reaction mixture containing Sirt1 (0.5 µM), **3** (20 µM), NAD^+^ (500 µM) and different concentrations of resveratrol (0-100 µM) in assay-buffer were incubated for 60 minutes at 37°C. Reaction was stopped using TFA (1% final concentration) and the consumption of **3** was analyzed using HPLC. Data represent average ± s.d. (n=3).

## Single Fluorescence Measurements

### Control Measurements

|  |
| --- |

**Supplementary Figure S5.** Fluorescence change as a function of time. The reaction were performed with 0.1 µM Sirt3, 500 µM NAD^+^ and 25µM peptide 3 (⚫) as well as either without NAD^+^ (⚫) or without Sirt3 (⚫).

**Supplementary Figure S6.** Fluorescence change as a function of enzyme concentration. The reactions were performed with 0.1, 0.2, 0.3 or 0.4 µM Sirt3, 500 µM NAD^+^ and 2µM peptide **3**. Data represent average ± s.d. (n=2) and lines are presented as linear regression.

**Supplementary Figure S7.**Progress curves of substrate turnover of peptide **3** by Sirt3 (0.1 µM). The reactions were performed with 500 µM NAD^+^ and ⚫ 0.5 µM, ⚫ 2 µM, ⚫ 5 µM, ⚫25 µM peptide **3**.

### Determination of kinetic constants

| **Sirt2 – peptide 4** | **Sirt2 – peptide 5** |
| --- | --- |
| **Sirt2 – peptide 4a** | **Sirt3 – peptide 3** |
| **Sirt4 – peptide 6** | **Sirt6 – peptide 3** |

**Supplementary Figure S8.** Kinetic characteristics of **3**, **4**, **5,** **6** and **4a** for different sirtuins. The reactions were performed with 0.01 µM Sirt2 /0.1 µM Sirt 3 /1 µM Sirt4 /0.5 µM Sirt6, 500 µM NAD^+^ and varying concentrations of indicated peptide (0.5-100 µM). The reactions were analyzed by fluorescence spectrophotometer. Data are presented as mean ± s.d. (n=2) and lines represent the Michaelis-Menten-plot.

### Calibration Lines

| **peptide 3** | **peptide 4** |
| --- | --- |
| **peptide 5** | **peptide 4a** |
| **peptide 6** |  |

**Supplementary Figure S9.** Calibration lines for peptides **3**, **4, 4a**, **5** and **6**. The reaction mixtures contained 2 µM Sirt2, 500 µM NAD^+^ and 100 µM of **3**, **4,** **4a** or **5**. After complete turnover of peptide substrate, the mixtures were diluted (0.1-25 µM) and analyzed by fluorescence measurements. Data represent mean ± s.d. (n=2) and lines represent linear regression.

### Simultaneous measurments of sirtuin activity using two substrates





**Supplementary Figure S10.** Excitation and emission spectra of peptides **3** and **6**. Reaction mixture containing **3** and **6** was excited at 290 ± 5 nm **(A)**. Emission spectra **(B)** of the different fluorophores enable simultaneous detection of sirtuin mediated deacylation at 408 nm and 535 nm for **3** and **6**, respectively.





**Supplementary Figure S11.**Simultaneous measurments of sirtuin activity using two substrates. Reaction mixtures contained Sirt4 (0.5 µM), **3** (10 µM), 6 (10 µM) and NAD^+^ (500 µM) **(A)** or Sirt2 (0.01 µM), **3** (1 µM), **6** (1 µM) and NAD^+^ (500 µM) **(B)**. Reactions were incubated in assay-buffer at 37°C. Fluorophores were excited at 290 ± 5 nm and emission spectra (350-550 nm) were recorded over time. Fluorescence intensities at 408 nm and 535 nm (maximum of emissions specific for substrates **3** and **6,** respectively) were extracted and plotted as a function of time. Data represent average ± s.d. (n=2).

## Microtiter plate fluorescence measurements

### Determination of kinetic constants

| **Sirt1**  **A** | **Sirt4**  **A** |
| --- | --- |
| **Sirt2**  **A** | **Sirt2**  **B** |
| **Sirt3**  **A** | **Sirt3**  **B** |
| **Sirt6**  **A** | **Sirt6**  **B** |

**Supplementary Figure S12.** Kinetic characteristics of **3** **(A)** and NAD^+^ **(B)** for different sirtuins. The reactions were performed 0.5 µM Sirt1 / 0.01 µM Sirt2 / 0.1 µM Sirt3 / 1 µM Sirt4 / 0.5 µM Sirt6 and either **(A)** 500 µM NAD^+^ and varying concentrations of **3** (0.07-100 µM) or **(B)** 10-1500 µM NAD^+^ and fixed concentrations of **3** (5, 25 or 200 µM). The reactions were analyzed by microplate fluorescence measurements. Data are presented as mean ± s.d. (n=2) and lines represent the Michaelis-Menten-plot.

### Determination of the inhibitor constant K_i_

| **Sirt3**  **A** | **** |
| --- | --- |
| **Sirt3**  **B** | **** |
| **Sirt6**  **A** | **** |
| **Sirt6**  **B** | **** |

**Supplementary Figure S13.** Kinetic characteristics for sirtuin inhibitor NAM. The reactions were performed with 0.1 µM Sirt 3 (3)/0.5 µM Sirt6 (6) and either **(A)** 500 µM NAD^+^ and varying concentrations of **3** (0.5-100 µM) or **(B)** 10-1500 µM NAD^+^ and fixed concentrations of **3** (25 or 200 µM). For determination of the K_i_ values different concentrations of inhibitor were used (● 0, ○ 25, ▼ 50, ▽ 75, ⯁ 100, ◇ 125, □ 150, ▲ 300, △ 450, 🟊 600 µM). Data are presented as mean ± s.d. (n=2) and lines represent the Michaelis-Menten-plot (left) and secondary plots (right). The apparent K_M_ or V_max_^app-1^ values from v/[S] characteristics were plotted against the corresponding inhibitor concentration. The negative K_i_ value can be determined as intersection with the X-axis from the K_M_^app^/I plot respectively from the V_max_^app-1^/I plot.

| **Ex-527** |  |
| --- | --- |
| **Quercetin** |  |

**Supplementary Figure S14.** Kinetic characteristics for sirtuin inhibitor Ex-527 and Quercetin. The reactions were performed with 0.5 µM Sirt6 and either 500 µM NAD^+^ and varying concentrations of **3** (2-100 µM). For determination of the K_i_ values different concentrations of inhibitor were used (● 0, ○ 10, ▼ 20, ▽ 40, ⯁ 60, ◇ 80 µM). Data are presented as mean ± s.d. (n=2) and lines represent the Michaelis-Menten-plot (left) and secondary plots (right). The apparent V_max_^app-1^ values from v/[S] characteristics were plotted against the corresponding inhibitor concentration. The negative K_i_ value can be determined as intersection with the X-axis from the V_max_^app-1^/I plot.

| **Sirt2** | **** |
| --- | --- |
| **Sirt3** | **** |
| **Sirt6** | **** |

**Supplementary Figure S15.** Kinetic characteristics for sirtuin inhibitor **8**. The reactions were performed with 0.01 µM Sirt2 /0.1 µM Sirt3 /0.5 µM Sirt6 and 500 µM NAD^+^ and varying concentrations of **3** (0.07-100 µM). For determination of the K_i_ values different concentrations of inhibitor were used (● 0, ○ 10, ▼ 20, ▽ 50, ⯁ 75, ◇ 100, ■ 150, □ 200, ▲ 350, △ 500 nM). Data are presented as mean ± s.d. (n=2) and lines represent the Michaelis-Menten-plot (left) and secondary plots (right). The apparent V_max_^app-1^ values from v/[S] characteristics were plotted against the corresponding inhibitor concentration. The negative K_i_ value can be determined as intersection with the X-axis from the V_max_^app-1^/I plot.

| **peptide 10** |  |
| --- | --- |
| **peptide 11** |  |

**Supplementary Figure S16.** Kinetic characteristics for sirtuin inhibitor **10** and **11**. The reactions were performed with 0.01 µM Sirt2 and 500 µM NAD^+^ and varying concentrations of **3** (0.07-5 µM). For determination of the K_i_ values different concentrations of inhibitor were used (**10** = ● 0, ○ 0.05, ▼ 0.1, ▽ 0.15, ⯁ 0.2 µM, **11** = ● 0, ○ 10, ▼ 20, ▽ 30, ⯁ 50 µM). Data are presented as mean ± s.d. (n=2) and lines represent the Michaelis-Menten-plot (left) and the secondary plots (right). The apparent V_max_^app-1^ values from v/[S] characteristics were plotted against the corresponding inhibitor concentration. The negative K_i_ value can be determined as intersection with the X-axis from the V_max_^app-1^/I plot.

| **** | **** |
| --- | --- |

**Supplementary Figure S17.** Kinetic characteristics for sirtuin inhibitor S2iL5. The reactions were performed with 0.01 µM Sirt2 and 500 µM NAD^+^ and varying concentrations of **3** (0.07-5 µM). For determination of the K_i_ values different concentrations of inhibitor were used (● 0, ○ 100, ▼ 200, ▽ 300, ⯁ 450, ◇ 600 nM). Data are presented as mean ± s.d. (n=2) and lines represent the Michaelis-Menten-plot (left) and secondary plots (right). The apparent V_max_^app-1^ values from v/[S] characteristics were plotted against the corresponding inhibitor concentration. The negative K_i_ value can be determined as intersection with the X-axis from the V_max_^app-1^/I plot.

### Calibration Curves

| **G182** | **G170** |
| --- | --- |
| **G160** |  |

**Supplementary Figure S18.** Calibration lines for **3**. The reaction mixtures contained 2 µM Sirt2, 500 µM NAD^+^ and 100 µM of **3**. After complete turnover of peptide substrate, the mixtures were diluted (0.1-25 µM) and analyzed by microplate reader at λ_Ex_ = 320 nm and λ_Em_ = 408 nm (lag time 9 µs, integration time 20 µM, gain 182 (G182), 170 (G170) and 160 (G160)). Data are presented as mean ± s.d. (n=2) and lines represent linear regression.

## Overview kinetic constants

**Supplementary Table S2.** Summary of kinetic constants for different peptide substrates and sirtuins.

| Compound | Method | Enzyme | K_M_^(peptide)^ [µM] | K_M_^(NAD+)^ [µM] | k_cat_ [s^-1^] | k_cat_/K_M_  [M^-1^s^-1^] |
| --- | --- | --- | --- | --- | --- | --- |
| **1a** | HPLC | Sirt2^[c]^ | 0.7 ± 0.1 | n.d. | 3.7∙10^-2^ ± 0.1∙10^-2^ | 5.30∙10^4^ |
|  |  | Sirt3^[c]^ | 1.6 ± 0.4 | n.d. | 4.7∙10^-2^ ± 0.3∙10^-2^ | 2.95∙10^4^ |
|  |  | Sirt5^[e]^ | 16.5 ± 3.1 | n.d. | 5.7∙10^-3^ ± 0.3∙10^-3^ | 3.48∙10^2^ |
|  |  | Sirt6^[e]^ | 5.7 ± 1.3 | n.d. | 4.7∙10^-3^ ± 0.3∙10^-3^ | 8.25∙10^2^ |
| **2a** | HPLC | Sirt6^[e]^ | 17.3 ± 2.6 | n.d. | 4.9∙10^-3^ ± 0.2∙10^-3^ | 2.81∙10^2^ |
| **3** | HPLC | Sirt2^[a]^ | 0.3 ± 0.04 | n.d. | 3.9∙10^-2^ ± 0.1∙10^-2^ | 1.18∙10^5^ |
|  |  | Sirt3^[c]^ | 3.3 ± 0.4 | n.d. | 1.0∙10^-2^ ± 0.3∙10^-3^ | 3.12∙10^3^ |
|  |  | Sirt5^[e]^ | 46.1 ± 7.2 | n.d. | 3.2∙10^-3^ ± 0.2∙10^-3^ | 69.00 |
|  |  | Sirt6^[e]^ | 18.3 ± 4.4 | n.d. | 8.3∙10^-4^ ± 0.7∙10^-4^ | 45.00 |
|  | FS | Sirt3^[c]^ | 2.4 ± 0.2 | n.d. | 1.2∙10^-2^ ± 0.3∙10^-3^ | 5.15∙10^3^ |
|  | MTPR | Sirt1^[e]^ | 0.7 ± 0.08 | n.d. | 2.1∙10^-3^ ± 0.6∙10^-4^ | 2.87∙10^2^ |
|  |  | Sirt2^[a]^ | 0.1 ± 0.02 | 33.0 ± 5.5 | 2.4∙10^-2^ ± 0.8∙10^-4^ | 1.76∙10^5^ |
|  |  | Sirt3^[c]^ | 3.3 ± 0.4 | 156.0 ± 24.1 | 9.1∙10^-3^ ± 0.4∙10^-3^ | 2.79∙10^3^ |
|  |  | Sirt4^[f]^ | 49.5 ± 7.5 | n.d. | 3.6·10^-4^ ± 0.2·10^-4^ | 7.00 |
|  |  | Sirt6^[e]^ | 23.5 ± 4.8 | 386.0 ± 56.0 | 9.1∙10^-4^ ± 0.6∙10^-4^ | 39.00 |
| **4** | HPLC | Sirt2^[e]^ | 26.8 ± 2.7 | n.d. | 3.9∙10^-3^ ± 0.2∙10^-3^ | 1.46∙10^2^ |
|  | FS |  | 17.7 ± 1.5 | n.d. | 4.0∙10^-3^ ± 0.1∙10^-3^ | 2.24∙10^2^ |
| **4a** | FS | Sirt2^[b]^ | 1.2 ± 0.1 | n.d. | 4.5∙10^-2^ ± 0.2∙10^-2^ | 3.86∙10^4^ |
| **5** | FS | Sirt2^[e]^ | 15.3 ± 2.7 | n.d. | 2.4∙10^-3^ ± 0.1∙10^-3^ | 1.56∙10^2^ |
| **6** | MTPR | Sirt2^[d]^ | 7.5 ± 1.5 | n.d. | 2.6∙10^-3^ ± 0.2∙10^-3^ | 3.42∙10^2^ |
| **7** | MTPR | Sirt2^[a]^ | 0.8 ± 0.1 | n.d. | 9.6∙10^-2^ ± 0.5∙10^-2^ | 1.25∙10^5^ |

[a] [E] = 0.01 µM; [b] [E] = 0.05 µM; [c] [E] = 0.1 µM; [d] [E] = 0.2 µM, [e] [E] = 0.5 µM, [f] [E] = 1 µM; n.d. - not determined; FS – fluorescence spectrometer; MTPR – microtiter plate reader. Data are presented as mean ± s.d. (n=2).

# Photo-induced change of *cis* content of thioxo peptides

## Temperature dependency of *cis*/*trans* isomerization

**284 nm**

**256 nm**

**A**

**B**

**C**

**D**

**Supplementary Figure S19.** Determination of *cis*/*trans* isomerization velocity of **8** at 10°C after 60 s irradiation. **(A)** UV-spectra in GS (black line), PSS (red line), from PSS to GS* after 1 h 15 min equilibration (green line) and GS* after 16 h (blue dots). **(B)** Differential spectrum. **(C)** Kinetic characterization of *cis*/*trans* isomerization from PSS to GS* at 256 nm (red line); equation used: A = A_0_ + a * (1 – e^(-kc/t*t)^); k_c/t_ = 2.33 x 10^-4^ s^-1^. **(D)** Kinetic characterization of *cis*/*trans* isomerization from PSS to GS* at 284 nm (red line); equation used: A = A_E_ + a * e^(-kc/t*t)^; k_c/t_ = 1.44 x 10^-4^ s^-1^.

 **Supplementary Figure S20.** Determination of *cis*/*trans* isomerization velocity of **8** at 20°C after 60 s irradiation. **(A)** UV-spectra in GS (black line), PSS (red line), from PSS to GS* after 30 min equilibration (green line) and GS* after 16 h (blue dots). **(B)** Differential spectrum. **(C)** Kinetic characterization of *cis*/*trans* isomerization from PSS to GS* at 256 nm (red line); equation used: A = A_0_ + a * (1 – e^(-kc/t*t)^); k_c/t_ = 5.04 x 10^-4^ s^-1^. **(D)** Kinetic characterization of *cis*/*trans* isomerization from PSS to GS* at 284 nm (red line) ; equation used: A = A_E_ + a * e^(-kc/t*t)^; k_c/t_ = 4.55 x 10^-4^ s^-1^.

**284 nm**

**256 nm**

**A**

**B**

**C**

**D**

**284 nm**

**256 nm**

**AS**

**B**

**C**

**D**

**Supplementary Figure S21.** Determination of *cis*/*trans* isomerization velocity of **8** at 30°C after 60 s irradiation. **(A)** UV-spectra in GS (black line), PSS (red line), from PSS to GS* after 15 min equilibration (green line) and GS* after 8 h (blue dots). **(B)** Differential spectrum. **(C)** Kinetic characterization of *cis*/*trans* isomerization from PSS to GS* at 256 nm (red line); equation used: A = A_0_ + a * (1 – e^(-kc/t*t)^); k_c/t_ = 1.16 x 10^-3^ s^-1^. **(D)** Kinetic characterization of *cis*/*trans* isomerization from PSS to GS* at 284 nm (red line); equation used: A = A_E_ + a * e^(-kc/t*t)^; k_c/t_ = 1.15 x 10^-3^ s^-1^.

**284 nm**

**256 nm**

**A**

**B**

**C**

**D**

**Supplementary Figure S22.** Determination of *cis*/*trans* isomerization velocity of **8** at 50°C after 60 s irradiation. **(A)** UV-spectra in GS (black line), PSS (red line), from PSS to GS* after 2 min equilibration (green line) and GS* after 1 h(blue dots). **(B)** Differential spectrum. **(C)** Kinetic characterization of *cis*/trans isomerization from PSS to GS* at 256 nm (red line); equation used: A = A_0_ + a * (1 – e^(-kc/t*t)^); k_c/t_ = 5.39 x 10^-3^ s^-1^. **(D)** Kinetic characterization of *cis*/*trans* isomerization from PSS to GS* at 284 nm (red line); equation used: A = A_E_ + a * e^(-kc/t*t)^; k_c/t_ = 5.03 x 10^-3^ s^-1^.

**284 nm**

**256 nm**

**A**

**B**

**C**

**D**

**Supplementary Figure S23.** Determination of *cis*/*trans* isomerization velocity of **8** at 70°C after 60 s irradiation. **(A)** UV-spectra in GS (black line), PSS (red line), from PSS to GS* after 1 min equilibration (green line) and GS* after 30 min (blue dots). **(B)** Differential spectrum. **(C)** Kinetic characterization of *cis*/*trans* isomerization from PSS to GS* at 256 nm (red line); equation used: A = A_0_ + a * (1 – e^(-kc/t*t)^); k_c/t_ = 1.03 x 10^-2^ s^-1^. **(D)** Kinetic characterization of *cis*/*trans* isomerization from PSS to GS* at 284 nm (red line); equation used: A = A_E_ + a * e^(-kc/t*t)^; k_c/t_ = 1.38 x 10^-2^ s^-1^.

**252 nm**

**278 nm**

**A**

**B**

**C**

**D**

**Supplementary Figure S24.** Determination of *cis*/*trans* isomerization velocity of **10** at 10°C after 50 s irradiation. **(A)** UV-spectra in GS (black line), PSS (red line), from PSS to GS* after 9 h equilibration (green line) and GS* after 16 h (blue dots). **(B)** Differential spectrum. **(C)** Kinetic characterization of *cis*/*trans* isomerization from PSS to GS* at 252 nm (red line); equation used: A = A_0_ + a * (1 – e^(-kc/t*t)^); k_c/t_ = 2.18 x 10^-5^ s^-1^. **(D)** Kinetic characterization of *cis*/*trans* isomerization from PSS to GS* at 278 nm (red line); equation used: A = A_E_ + a * e^(-kc/t*t)^; k_c/t_ = 2.04 x 10^-5^ s^-1^.

**278 nm**

**252 nm**

**A**

**B**

**C**

**D**

**Supplementary Figure S25.** Determination of *cis*/*trans* isomerization velocity of **10** at 20°C after 50 s irradiation. **(A)** UV-spectra in GS (black line), PSS (red line), from PSS to GS* after 3 h equilibration (green line) and GS* after 16 h (blue dots). **(B)** Differential spectrum. **(C)** Kinetic characterization of *cis*/*trans* isomerization from PSS to GS* at 252 nm (red line); equation used: A = A_0_ + a * (1 – e^(-kc/t*t)^); k_c/t_ = 6.59 x 10^-5^ s^-1^. **(D)** Kinetic characterization of *cis*/*trans* isomerization from PSS to GS* at 278 nm (red line); equation used: A = A_E_ + a * e^(-kc/t*t)^; k_c/t_ = 6.40 x 10^-5^ s^-1^.

**278 nm**

**252 nm**

**A**

**B**

**CV**

**D**

**Supplementary Figure S26.** Determination of *cis*/*trans* isomerization velocity of **10** at 30°C after 50 s irradiation. **(A)** UV-spectra in GS (black line), PSS (red line), from PSS to GS* after 1 h 30 min equilibration (green line) and GS* after 8 h (blue dots). **(B)** Differential spectrum. **(C)** Kinetic characterization of *cis*/*trans* isomerization from PSS to GS* at 252 nm (red line); equation used: A = A_0_ + a * (1 – e^(-kc/t*t)^); k_c/t_ = 1.57 x 10^-4^ s^-1^. **(D)** Kinetic characterization of *cis*/*trans* isomerization from PSS to GS* at 278 nm (red line); equation used: A = A_E_ + a * e^(-kc/t*t)^; k_c/t_ = 1.49 x 10^-4^ s^-1^.

**278 nm**

**252 nm**

**A**

**B**

**C**

**D**

**Supplementary Figure S27.** Determination of *cis*/*trans* isomerization velocity of **10** at 50°C after 50 s irradiation. **(A)** UV-spectra in GS (black line), PSS (red line), from PSS to GS* after 14 min equilibration (green line) and GS* after 1 h (blue dots). **(B)** Differential spectrum. **(C)** Kinetic characterization of *cis*/*trans* isomerization from PSS to GS* at 252 nm (red line); equation used: A = A_0_ + a * (1 – e^(-kc/t*t)^); k_c/t_ = 8.98 x 10^-4^ s^-1^. **(D)** Kinetic characterization of *cis*/*trans* isomerization from PSS to GS* at 278 nm (red line); equation used: A = A_E_ + a * e^(-kc/t*t)^; k_c/t_ = 7.08 x 10^-4^ s^-1^.

**278 nm**

**252 nm**

**A**

**B**

**C**

**D**

**Supplementary Figure S28.** Determination of *cis*/*trans* isomerization velocity of **10** at 70°C after 50 s irradiation. **(A)** UV-spectra in GS (black line), PSS (red line), from PSS to GS* after 2 min equilibration (green line) and GS* after 30 min (blue dots). **(B)** Differential spectrum. **(C)** Kinetic characterization of *cis*/*trans* isomerization from PSS to GS* at 252 nm (red line); equation used: A = A_0_ + a * (1 – e^(-kc/t*t)^); k_c/t_ = 2.75 x 10^-3^ s^-1^. **(D)** Kinetic characterization of *cis*/*trans* isomerization from PSS to GS* at 278 nm (red line); equation used: A = A_E_ + a * e^(-kc/t*t)^; k_c/t_ = 4.73 x 10^-3^ s^-1^.

**255 nm**

**280 nm**

**A**

**B**

**C**

**D**

**Supplementary Figure S29.** Determination of *cis*/*trans* isomerization velocity of **11** at 10°C after 45 s irradiation. **(A)** UV-spectra in GS (black line), PSS (red line), from PSS to GS* after 1 h 30 min equilibration (green line) and GS* after 16 h (blue dots). **(B)** Differential spectrum. **(C)** Kinetic characterization of *cis*/*trans* isomerization from PSS to GS* at 255 nm (red line); equation used: A = A_0_ + a * (1 – e^(-kc/t*t)^); k_c/t_ = 1.44 x 10^-4^ s^-1^. **(D)** Kinetic characterization of *cis*/*trans* isomerization from PSS to GS* at 280 nm (red line); equation used: A = A_E_ + a * e^(-kc/t*t)^; k_c/t_ = 1.26 x 10^-4^ s^-1^.

**280 nm**

**255 nm**

**A**

**B**

**C**

**D**

**Supplementary Figure S30.** Determination of *cis*/*trans* isomerization velocity of **11** at 20°C after 45 s irradiation. **(A)** UV-spectra in GS (black line), PSS (red line), from PSS to GS* after 45 min equilibration (green line) and GS* after 16 h (blue dots). **(B)** Differential spectrum. **(C)** Kinetic characterization of *cis*/*trans* isomerization from PSS to GS* at 255 nm (red line); equation used: A = A_0_ + a * (1 – e^(-kc/t*t)^); k_c/t_ = 2.75 x 10^-4^ s^-1^. **(D)** Kinetic characterization of *cis*/*trans* isomerization from PSS to GS* at 280 nm (red line); equation used: A = A_E_ + a * e^(-kc/t*t)^; k_c/t_ = 2.24 x 10^-4^ s^-1^.

**A**

**B**

**C**

**D**

**280 nm**

**255 nm**

**Supplementary Figure S31.** Determination of *cis*/*trans* isomerization velocity of **11** at 30°C after 45 s irradiation. **(A)** UV-spectra in GS (black line), PSS (red line), from PSS to GS* after 15 min equilibration (green line) and GS* after 8 h (blue dots). **(B)** Differential spectrum. **(C)** Kinetic characterization of *cis*/*trans* isomerization from PSS to GS* at 255 nm (red line); equation used: A = A_0_ + a * (1 – e^(-kc/t*t)^); k_c/t_ = 7.19 x 10^-4^ s^-1^. **(D)** Kinetic characterization of *cis*/*trans* isomerization from PSS to GS* at 280 nm (red line); equation used: A = A_E_ + a * e^(-kc/t*t)^; k_c/t_ = 6.25 x 10^-4^ s^-1^.

**280 nm**

**255 nm**

**A**

**B**

**C**

**D**

**Supplementary Figure S32.** Determination of *cis*/*trans* isomerization velocity of **11** at 50°C after 45 s irradiation. **(A)** UV-spectra in GS (black line), PSS (red line), from PSS to GS* after 4 min equilibration (green line) and GS* after 1 h (blue dots). **(B)** Differential spectrum. **(C)** Kinetic characterization of *cis*/*trans* isomerization from PSS to GS* at 255 nm (red line); equation used: A = A_0_ + a * (1 – e^(-kc/t*t)^); k_c/t_ = 3.67 x 10^-3^ s^-1^. **(D)** Kinetic characterization of *cis*/*trans* isomerization from PSS to GS* at 280 nm (red line); equation used: A = A_E_ + a * e^(-kc/t*t)^; k_c/t_ = 3.17 x 10^-3^ s^-1^.

**280 nm**

**255 nm**

**A**

**B**

**C**

**D**

**Supplementary Figure S33.** Determination of *cis*/*trans* isomerization velocity of **11** at 70°C after 45 s irradiation. **(A)** UV-spectra in GS (black line), PSS (red line), from PSS to GS* equilibration (green line) and GS* after 1 h (blue dots). **(B)** Differential spectrum. **(C)** Kinetic characterization of *cis*/*trans* isomerization from PSS to GS* at 255 nm (red line); equation used: A = A_0_ + a * (1 – e^(-kc/t*t)^); k_c/t_ = 9.50 x 10^-3^ s^-1^. **(D)** Kinetic characterization of *cis*/*trans* isomerization from PSS to GS* at 280 nm (red line) ; equation used: A = A_E_ + a * e^(-kc/t*t)^; k_c/t_ = 2.34 x 10^-2^ s^-1^.

**Supplementary Table S3.** Half-life values of *cis*/*trans* isomerization of **8**, **10** and **11** at different temperatures.

|  | **half-life t_1/2_ [min]** | | | | |
| --- | --- | --- | --- | --- | --- |
|  | **10 °C** | **20 °C** | **30 °C** | **50 °C** | **70 °C** |
| **8** | 61.3 | 24.1 | 10.0 | 2.2 | 1.0 |
| **10** | 547.5 | 178.0 | 78.5 | 14.4 | 3.1 |
| **11** | 85.6 | 46.4 | 17.2 | 3.4 | 0.7 |

**Supplementary Table S4.** Activation parameter of *cis*/*trans* isomerization of **8**, **10** and **11**.

|  | **ΔH^‡^ [kJ/mol]** | **ΔS^‡^ [J/mol*K]** | **ΔG^‡^ [kJ/mol]^[a]^** |
| --- | --- | --- | --- |
| **8** | 55.73 ± 3.82 | -118.73 ± 18.75 | 89.33 |
| **10** | 66.32 ± 0.96 | -99.34 ± 3.15 | 94.43 |
| **11** | 63.51 ± 2.64 | -95.78 ± 8.11 | 90.62 |

[a] determined using Gibbs-Helmholtz-equation: ΔG^‡^ = ΔH^‡^ - T*ΔS^‡^; T = 283 K

## Determination of *cis*/*trans* content

**Supplementary Table S5.** Content of *cis*-isomer of **9** in different solvents.

| **solvent** | **content of *cis*-isomer [%]** |
| --- | --- |
| assay-buffer | 43.3 |
| acetic acid | 51.0 |
| TFA | 40.6 |
| trifluoro ethanol (TFE) | 40.1 |
| 0.5 M LiCl/H_2_O | 42.4 |
| 0.5 M LiCl/EtOH | 51.9 |
| 0.5 M LiCl/TFE | 37.8 |
| methanol | 49.5 |
| formic acid | 44.6 |
| N-methyl pyrolidon (NMP) | 58.1 |
| DMF | 55.4 |
| DMSO | 45.8 |
| THF | 54.2 |

**Supplementary Figure S34.** Determination of *cis*/*trans* ratio in GS and PSS after 5 min irradiation at 254 nm and GS* after 30 min equilibration at 50 °C of **10** (A), **11** (B) and **8** (C).

## Separation of isomers

**A**

**B**

**Supplementary Figure S35.** Determination of isomerization velocity of of **9**. Isomerization velocity of a 30 µM solution was determined after preparative separation of *cis*/*trans* isomers at 20 °C. **(A)** UV-spectrum at start conditions (black line), after 15 min incubation (red line) and after 60 min incubation (green line); small figure shows differential spectrum. **(B)** Kinetic characteristic of isomerization. Data are presented as mean ± s.d. (n=3) and were fitted to A = A_0_ + a * (1 – e^(-kc/t*t)^); k_c/t_ = 9.50 x 10^-3^ s^-1^.

## Determination of isomer specific inhibition of sirtuins by thioxo peptides

**Supplementary Figure S36.** Determination of IC_50_ value of **8** for Sirt6 in GS (●) and PSS (▲). Data were fitted with U_(%)_ = U_min(%)_ + (U_max(%)_ – U_min(%)_)/(1+10^(x-logIC50)^) with U_(%)_ percental turmover of substrate **2a**; U_min(%)_ lowest turnover of substrate; U_max(%)_ maximal turnover of substrate (n=3).

**Supplementary Figure S37.** Inhibition of Sirt6 by **8** (**●**) and **9** (**▲**).Data were fitted with U_(%)_ = U_min(%)_ + (U_max(%)_ – U_min(%)_)/(1+10^(x-logIC50)^) with U_(%)_ percental turmover of substrate; U_min(%)_ lowest turnover of substrate **2a**; U_max(%)_ maximal turnover of substrate (n=3).

**Supplementary Figure S38.** Determination of IC_50_ of **9** for Sirt6 with 72.4 % *cis*- (▲) and 70.3 % (●) *trans*-content. Measurements were done with 10 µM peptide substrate (big figure) or 30 µM (small figure). Data were fitted with U_(%)_ = U_min(%)_ + (U_max(%)_ – U_min(%)_)/(1+10^(x-logIC50)^) with U_(%)_ percental turmover of substrate **2a**; U_min(%)_ lowest turnover of substrate; U_max(%)_ maximal turnover of substrate (n=3).

**Supplementary Figure S39.** Stability of separated isomers of **9** at a storage temperature of -70 °C for 2 weeks. Shown is the decrease of *cis*-isomer (●; k_iso_ = 0.39 ± 0.11 d^-1^) of fraction 3 and increase of *cis*-isomer (▲;k_iso_ = 0.26 ± 0.08 d^-1^) of fraction 7.

# NMR spectra


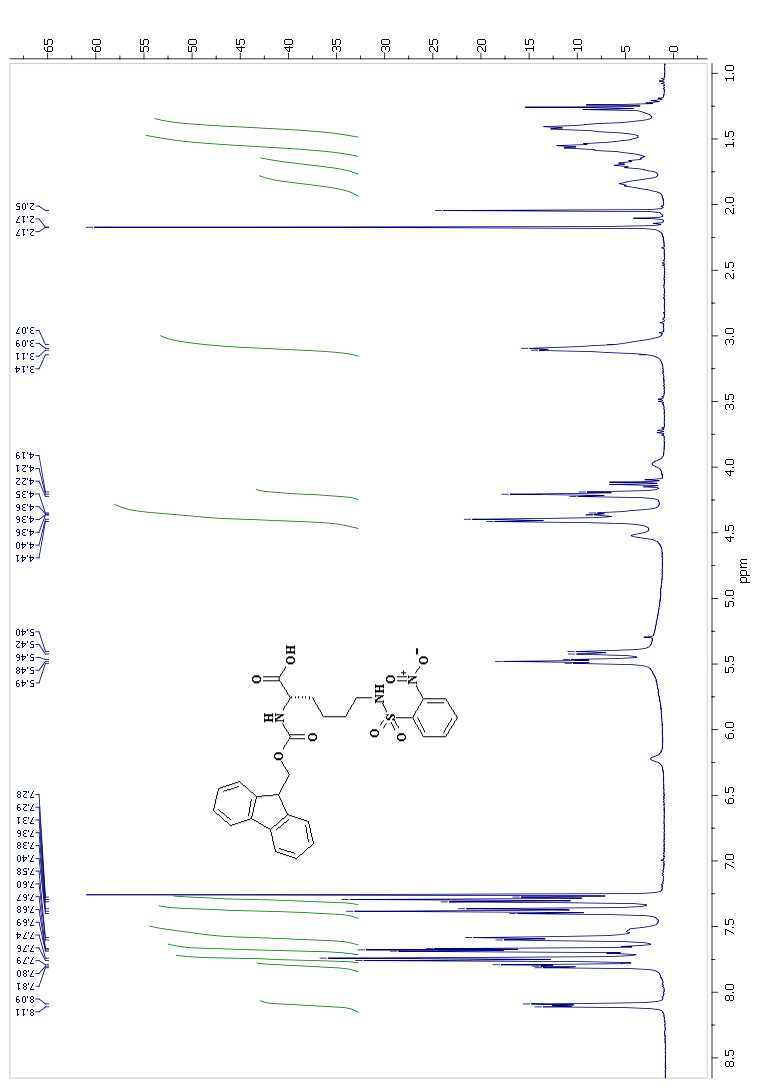


**Supplementary Figure S40.** ^1^H-NMR-spectra of Fmoc-Lys(Ns)-OH.


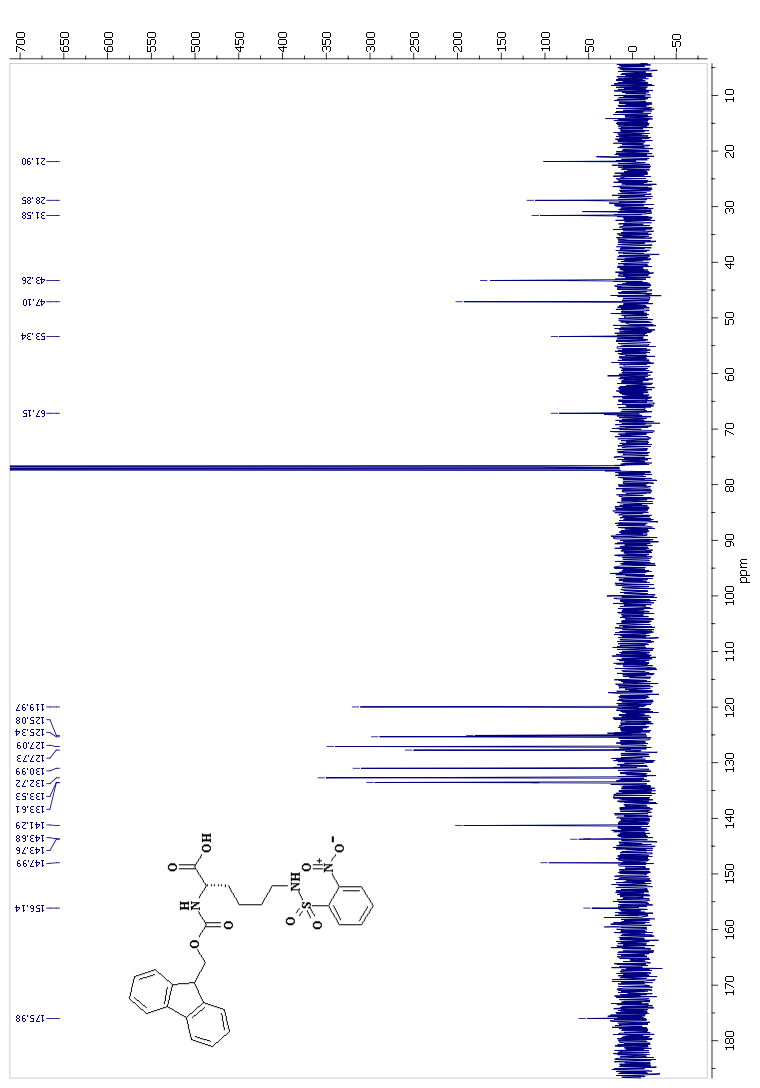


**Supplementary Figure S41.** ^13^C-NMR-spectra of Fmoc-Lys(Ns)-OH

# HPLC chromatograms and MS spectra of peptide derivatives

| **a** | **b** |
| --- | --- |

**Supplementary Figure S42**. Analytical HPLC (**a**) and MS-spectrum (**b**) of peptide **1b.**

| **a** | **b** |
| --- | --- |

**Supplementary Figure S43**. Analytical HPLC (**a**) and MS-spectrum (**b**) of peptide **2a**.

| **a** | **b** |
| --- | --- |

**Supplementary Figure S44**. Analytical HPLC (**a**) and MS-spectrum (**b**) of peptide **2b**.

| **a** | **b** |
| --- | --- |

**Supplementary Figure S45**. Analytical HPLC (**a**) and MS-spectrum (**b**) of peptide **3**.

| **a** | **b** |
| --- | --- |

**Supplementary Figure S46**. Analytical HPLC (**a**) and MS-spectrum (**b**) of peptide **4**.

| **a** | **b** |
| --- | --- |

**Supplementary Figure S47**. Analytical HPLC (**a**) and MS-spectrum (**b**) of peptide **5**.

| **a** | b |
| --- | --- |

**Supplementary Figure S48**. Analytical HPLC (**a**) and MS-spectrum (**b**) of peptide **6**.

| **a** | **b** |
| --- | --- |

**Supplementary Figure S49**. Analytical HPLC (**a**) and MS-spectrum (**b**) of peptide **7**.

|  |  |
| --- | --- |

**Supplementary Figure S50**. MS-spectrum of peptide **8**.

|  |  |
| --- | --- |

**Supplementary Figure S51.** MS-spectrum of peptide **10**.

| **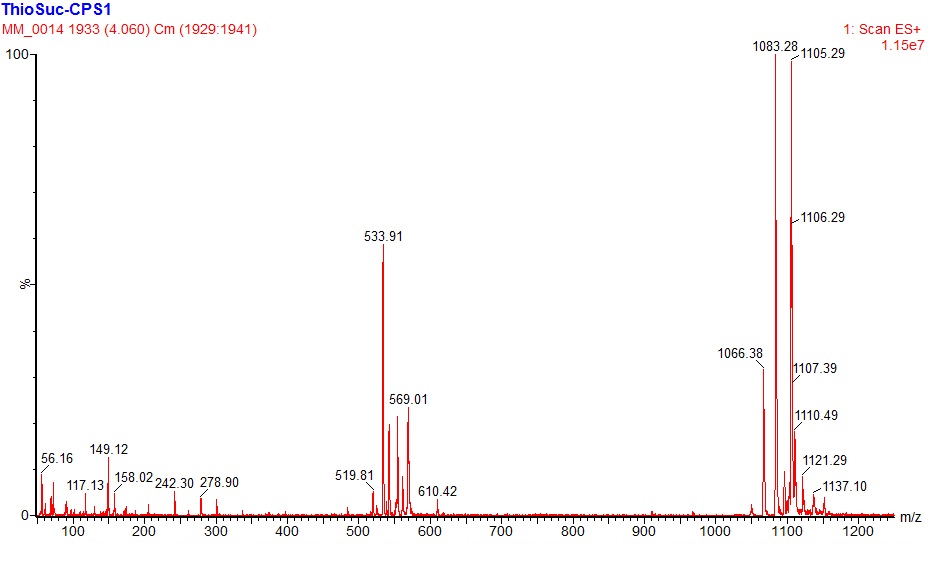** |  |
| --- | --- |

**Supplementary Figure S52.** MS-spectrum of peptide **11**.

| **a** | **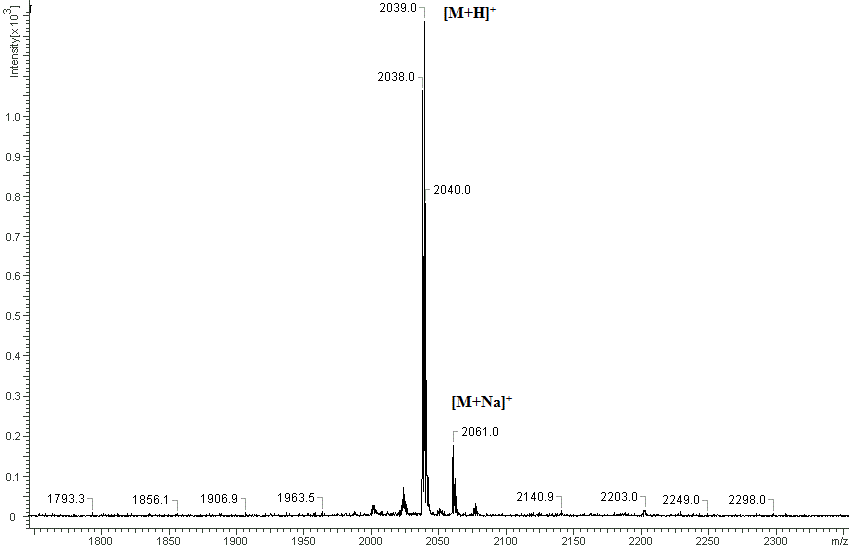b** |
| --- | --- |

**Supplementary Figure S53.** Analytical HPLC (**a**) and MS-spectrum (**b**) of peptide S2iL5.

| **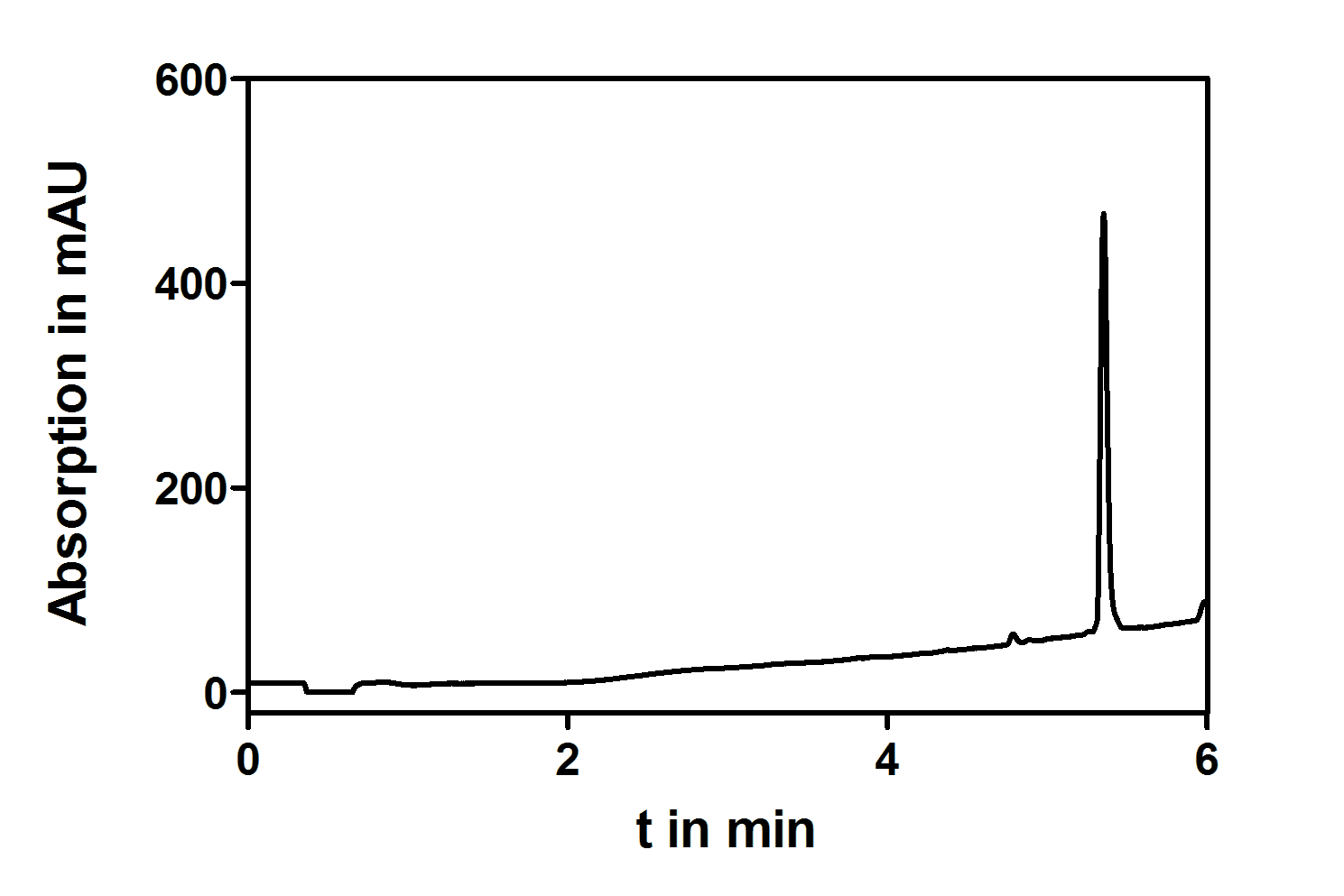a** | **b** |
| --- | --- |

**Supplementary Figure S54.** Analytical HPLC (**a**) and MS-spectrum (**b**) of peptide **1a**.
